# A Comprehensive Review of Catalytic Hydrodeoxygenation of Lignin-Derived Phenolics to Aromatics

**DOI:** 10.3390/molecules30102225

**Published:** 2025-05-20

**Authors:** Sitong Dong, Gang Feng

**Affiliations:** School of Chemical Engineering and Technology, Hebei University of Technology, Tianjin 300130, China

**Keywords:** lignin, hydrodeoxygenation, phenolic compounds, benzene, aromatic hydrocarbon, electrocatalytic hydrogenation

## Abstract

Single-ring aromatic compounds including BTX (benzene, toluene, xylene) serve as essential building blocks for high-performance fuels and specialty chemicals, with extensive applications spanning polymer synthesis, pharmaceutical manufacturing, and aviation fuel formulation. Current industrial production predominantly relies on non-renewable petrochemical feedstocks, posing the dual challenges of resource depletion and environmental sustainability. The catalytic hydrodeoxygenation (HDO) of lignin-derived phenolic substrates emerges as a technologically viable pathway for sustainable aromatic hydrocarbon synthesis, offering critical opportunities for lignin valorization and biorefinery advancement. This article reviews the relevant research on the conversion of lignin-derived phenolic compounds’ HDO to benzene and aromatic hydrocarbons, systematically categorizing and summarizing the different types of catalysts and their reaction mechanisms. Furthermore, we propose a strategic framework addressing current technical bottlenecks, highlighting the necessity for the synergistic development of robust heterogeneous catalysts with tailored active sites and energy-efficient process engineering to achieve scalable biomass conversion systems.

## 1. Introduction

The progressive depletion of conventional hydrocarbon reserves coupled with escalating ecological degradation necessitates paradigm-shifting innovations in sustainable energy systems. Within this context, lignocellulosic biomass has emerged as a strategic carbon-neutral feedstock, with its cascade conversion technologies attracting concerted research efforts across global scientific and industrial communities. Particularly, the inherent aromaticity of lignin derivatives positions them as biogenic precursors for synthetic aromatics’ production—a critical pathway for circular carbon economy implementation [1,2,3,4].

**Figure 1:**
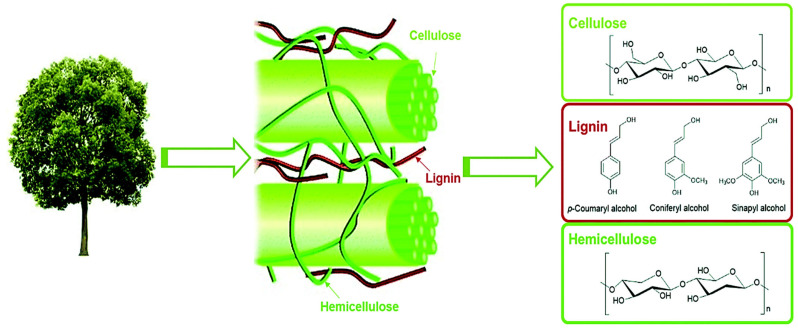
The schematic diagram of the composition of lignocellulosic biomass [23]. Copyright 2016 Royal Society of Chemistry.

As the predominant biomass reservoir on Earth, lignocellulosic materials yield an annual global output exceeding 170 billion metric tons [5,6,7,8]. Structurally characterized by a heterogeneous macromolecular matrix, these materials are constituted by three interwoven biopolymers: (1) cellulose (30–50%), a crystalline glucose polymer; (2) hemicellulose (20–30%), an amorphous heteropolysaccharide; and (3) lignin (15–30%), a methoxylated phenylpropanoid network (Figure 1) [9,10,11]. The structurally well-defined carbohydrate polymers (cellulose/hemicellulose) have been extensively valorized through established biorefinery protocols, serving as feedstocks for second-generation bioethanol and platform chemicals [12,13,14,15,16]. Conversely, lignin’s highly branched polyphenolic architecture—featuring C-C/C-O-C interunit linkages—creates a persistent technological bottleneck for selective depolymerization [1,4,17]. This structural recalcitrance confines over 98% of industrial lignin to low-value thermal applications [18,19,20], where its combustion not only exhibits suboptimal energy efficiency (~50% lower calorific value than coal) but also exacerbates carbon sequestration deficits through non-circular CO_2_ emissions [21,22]. Such systemic underutilization underscores the imperative for advanced catalytic strategies to unlock lignin’s latent potential as a renewable aromatic reservoir.

Contemporary lignin valorization strategies predominantly employ a two-stage cascade biorefinery paradigm: initial depolymerization of the macromolecular matrix into phenolic monomers, followed by precision HDO catalysis to yield premium aromatic hydrocarbons [24,25]. Mechanistic studies reveal two competing reaction coordinates in phenolic HDO processes: (i) a C-O bond scission with aromatic ring preservation via selective hydrogenolysis and (ii) ring hydrogenation concurrent with oxygen removal. The former pathway proves particularly advantageous for synthesizing drop-in fuel components, given aromatic hydrocarbons’ superior octane ratings (e.g., toluene: ~120 RON) and direct compatibility with existing fuel infrastructure [26]. Notably, the aromatic-selective routes demonstrate enhanced process economics, with hydrogen consumption metrics reduced by 30–50% compared to saturation pathways—a critical efficiency parameter for industrial scalability.

The technological impasse resides in achieving kinetically favored C-O cleavage over thermodynamically preferential C-C bond rupture. This selectivity conundrum necessitates atomic-level catalyst engineering to manipulate adsorption configurations and transition-state energetics. Recent breakthroughs in adsorption configuration engineering (e.g., tilted vs. flat phenol adsorption modes) and electronic perturbation strategies (metal–support charge-transfer effects) have opened new frontiers in aromatic yield optimization [27,28].

Improving the selectivity and yield of phenol HDO for the preparation of BTX (benzene, toluene, xylene) faces multiple challenges, with the core issue being how to precisely regulate the dynamic competition between catalyst active sites and reaction pathways [29,30,31,32]. Firstly, catalyst designs need to balance the dual-functional requirements of deoxygenation and aromatic ring stabilization: metal sites need to provide moderate hydrogenation capacity to avoid oversaturation of aromatic rings, while acidic supports need to promote deoxygenation but avoid strong acid-induced side reactions. Secondly, selective control of the reaction pathway is particularly crucial. Phenol HDO may be carried out through direct deoxygenation or hydrogenation dehydration pathways. The former directly generates aromatic hydrocarbons but requires inhibition of the deep hydrogenation of intermediates such as cyclohexanone, while the latter can easily lead to aromatic ring saturation and reduce BTX yield. In addition, the complexity of lignin-derived phenolic substrates, such as substituent positions and methoxy steric hindrance, can significantly interfere with deoxygenation efficiency.

Accordingly, a great deal of exceedingly excellent articles and reviews concerning the HDO of lignin-derived phenolic compounds have been published due to the importance of the HDO reaction in biomass conversion and upgradation. Seminal reviews by Zhong et al. established the methodological compendium for phenolic HDO across heterogeneous, homogeneous, and enzymatic systems [33]. Subsequent analyses by Wang’s team systematically deconstructed the multifunctional synergy in metal–acid bifunctional catalysts [34], while Sreedhar’s work mapped the reaction landscape for cycloalkane intermediates’ production [35]. Collectively, these intellectual foundations provide the crucial guides required for next-generation catalyst architectures [2,33,34,35].

At present, a large number of excellent articles have been published on the conversion of phenolic compounds’ HDO into aromatic hydrocarbons, among which benzene, toluene, and xylene (BTX) are three typical aromatic compounds [36]. In the current research field, there are few systematic reviews on the development of catalysts for the selective HDO of phenols to prepare aromatic hydrocarbons. In view of this, we did not focus on precious metal/non-precious metal catalysts or general lignin conversion pathways as in previous reviews but comprehensively reviewed the latest research results on the catalytic system and related strategies of lignin-derived phenolic compounds for the sustainable production of aromatic hydrocarbons through HDO catalysis. At the same time, a summary and explanation of the reaction pathways and mechanisms involved were provided to help deepen the understanding of the basic characteristics of catalysts. Finally, we put forward some preliminary suggestions for future research, hoping to promote the industrialization process of synthesizing high-value-added aromatic hydrocarbons and other chemicals from renewable lignin resources.

## 2. Catalytic Conversion of Phenolics into Aromatics

Benzene–toluene–xylene (BTX) constitutes the principal aromatic triad in petrochemical manufacturing, commanding an annual global output exceeding 10,000 tons through conventional naphtha reforming and steam-cracking processes [37]. They are currently used in a wide range of applications. In addition to being used as fuel additives and solvents, they can also be considered as the starting materials for the manufacture of various chemicals and polymers [25,26,37,38,39,40,41]. With escalating decarbonization mandates, biogenic BTX production via lignin-derived phenolic HDO has emerged as a carbon-negative alternative to fossil-derived routes—demonstrating lower lifecycle greenhouse gas emissions [42]. The evolving frontier in catalyst engineering has witnessed remarkable progress in designing heterogeneous catalytic systems for aromatic compound production. Advanced material platforms spanning loaded metal to transition metal compounds (sulfides, carbides, nitrides, phosphides) now demonstrate tailored efficacy in transforming lignocellulosic phenolic derivatives into benzene–toluene–xylene (BTX). This diversification in catalytic design strategies enables precise control over deoxygenation pathways and aromatic stabilization mechanisms.

### 2.1. Noble Metal Catalysts

Noble metals dominate phenolic HDO research due to their unparalleled hydrogen dissociation efficiency and oxygen-removal capabilities. Currently, three major noble metals, including palladium, platinum, and ruthenium, were studied extensively and considered as the most suitable metal catalysts in the BTX production from lignin-derived phenolic compounds.

#### 2.1.1. Palladium (Pd)-Based Catalysts

**Figure 2:**
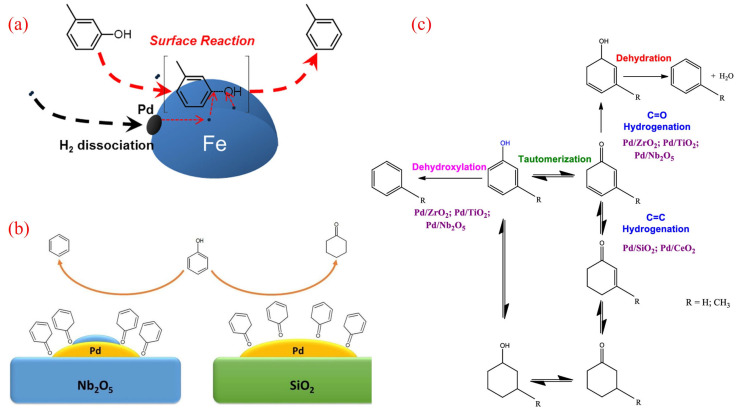
(**a**) Mechanism of Pd-Fe synergy in HDO of m-cresol [43]. Copyright 2014 American Chemical Society. (**b**) The HDO reaction of phenol over Pd/SiO_2_ and Pd/Nb_2_O_5_ [44]. Copyright 2017 Elsevier. (**c**) Reaction scheme for the HDO of phenolics over Pd supported on various oxides [47]. Copyright 2021 American Chemical Society.

Palladium (Pd), a precious and costly metal, plays a significant role in the hydrodeoxygenation (HDO) of phenolics. Hong et al. explored the synergistic catalytic effects of Pd and Fe in the HDO of m-cresol using Pd/Fe_2_O_3_ catalysts [43]. Their study revealed that the addition of Pd enhances the reduction of Fe under an H_2_ atmosphere, and the reduced catalyst demonstrates resistance to the Fe surface oxidation caused by reactants and water during the HDO process. Through characterization and kinetic studies, the synergistic effect between Pd and Fe was identified, being attributed to three key factors: the promotion of H_2_ activation by Pd, the stabilization of metallic Fe, and the enhancement of product desorption by Pd. The mechanism of Pd-Fe synergy is illustrated in Figure 2a. It involves H_2_ preferentially adsorbing and dissociating on Pd entities attached to the Fe surface, followed by spillover to metallic Fe sites where m-cresol adsorbs and activates [34]. The unique adsorption pattern of m-cresol on the Fe surface contributes to the high selectivity of BTX as direct HDO products. Additionally, Pd serves as the active site for H_2_ activation, maintaining high hydrogen coverage on the metallic Fe surface. These findings suggest that the proposed synergistic catalysis could be extended to other noble metal-promoted Fe catalysts, offering a promising strategy for designing highly active HDO catalysts in the future.

The choice of load material significantly influences both the activity and product distribution in HDO reactions. Barrios and colleagues investigated the performance of Pd loaded on SiO_2_ and Nb_2_O_5_ [44,45]. Their results showed that the reaction rate for phenol HDO over Pd/Nb_2_O_5_ was 90 times higher than that of the SiO_2_-loaded catalyst. Furthermore, the main product varied depending on the support: cyclohexanone was predominantly formed over Pd/SiO_2_, while benzene was the primary product over Pd/Nb_2_O (Figure 2b). The high activity and selectivity toward benzene for Pd/Nb_2_O were likely due to the strong interaction between the oxygenophilic Nb^5+^/Nb^4+^ cations and the oxygen in the phenol molecule [44]. Similarly, De Souza and colleagues compared the catalytic performance of Pd supported on various carriers, including SiO_2_, Al_2_O_3_, TiO_2_, ZrO_2_, CeO_2_, and CeZrO_2_, for phenol HDO [46]. For instance, benzene was the main product for Pd/TiO_2_ and Pd/ZrO_2_, whereas cyclohexanone predominated for Pd/SiO_2_, Pd/Al_2_O_3_, Pd/CeO_2_, and Pd/CeZrO_2_. The high selectivity toward benzene for the Pd/TiO_2_ and Pd/ZrO_2_ catalysts may be attributed to the oxophilic sites represented by the incompletely coordinated Ti^4+^ and Zr^4+^ cations near the metal particle periphery. In contrast, the weaker interaction between the metal cations of other supports and oxygen favored the hydrogenation of phenol to cyclohexanone.

Recent studies further explored the reaction pathways for the HDO of phenolics using Pd supported on different carriers in a fixed-bed reactor at 573 K [47]. The results demonstrated that the support type significantly influences the product distribution. For example, Pd supported on SiO_2_ and CeO_2_ favored ring hydrogenation, leading to oxidation products such as cyclohexanone. In contrast, the use of ZrO_2_, TiO_2_, and Nb_2_O_5_ as supports promoted the formation of benzene and toluene through either the carbonyl hydrogenation or direct deoxygenation of reciprocal isomeric intermediates (Figure 2c). Additionally, the reaction pathway for the removal of the methoxy group in the HDO of anisole was found to depend on the support. For all catalysts except Pd/Nb_2_O_5_, the preferred pathway involved demethylation to produce phenol, followed by further deoxygenation to benzene. However, the superior oxygenophilicity of Nb cations in Pd/Nb_2_O_5_ favored direct deoxygenation, resulting in the formation of benzene and methanol.

#### 2.1.2. Ruthenium (Ru)-Based Catalysts

Ruthenium-based catalysts have demonstrated exceptional performance in the hydrodeoxygenation (HDO) of lignin-derived phenolics, particularly in enhancing BTX (benzene, toluene, xylene) formation. A breakthrough study by Wang et al. revealed that Ru/Nb_2_O_5_ systems achieved 64% BTX selectivity with a 35.5% yield during 20 h organosolv lignin conversion, substantially outperforming Ru catalysts supported on ZrO_2_, Al_2_O_3_, and TiO_2_ [48]. Notably, this configuration exhibited 80% toluene selectivity in p-cresol conversion through optimized C-O bond scission. Mechanistic investigations combining spectroscopic characterization and density functional theory (DFT) analyses elucidated Nb_2_O_5_’s dual functionality: a strong phenol adsorption capacity and remarkable reduction in the activation energy for aromatic C-O cleavage. The catalytic synergy arises from Ru’s hydrogenolytic activity, where surface-adsorbed H* species facilitate aryl-O bond dissociation. Crucially, the weak π-complex interaction between the generated aromatics and Nb_2_O_5_ surface enables rapid product desorption, effectively suppressing overhydrogenation side reactions. This mechanistic framework aligns with recent advancements in Au-Nb_2_O_5_ nanocomposites, which demonstrated analogous C-O bond activation efficiency during lignin hydrogenolysis [30].

Solvent engineering significantly regulates the efficiency and product distribution of hydrogenolysis deoxygenation (HDO) reactions through key parameters such as solubility, polarity, and hydrogen supply capacity. Currently, biomass-catalyzed HDO mainly uses water, alcohols, and their composite solvent systems [49,50]. Based on this, the research team developed the Ru/Nb_2_O_5_-MC catalyst through an initial wet impregnation method and systematically investigated its solvent effect in phenolic compound HDO [51]. Under optimized conditions of 250 °C and 2-bar H_2_, the biphasic catalytic system achieved the complete conversion of phenol and obtained 80% benzene selectivity. Compared to a single decahydronaphthalene or aqueous system, the decahydronaphthalene/water biphasic system increases benzene selectivity by 15–20%. Its advantage lies in the metal–acid dual-functional synergistic mechanism: Ru nanoparticles activate hydrogen molecules through dissociation adsorption, while the acidic sites of the Nb_2_O_5_-MC carrier can specifically adsorb oxygen-containing intermediates, promoting deoxygenation pathways through polarized Caryl-OH bonds and stable dehydration transition states. The water phase at the biphasic interface effectively suppresses the benzene ring hydrogenation side reaction through thermodynamic regulation, resulting in a 30–40% increase in selectivity. The kinetic isotope effect experiment confirms that this process mainly follows the direct deoxygenation mechanism. This solvent-mediated catalytic strategy achieved the efficient synthesis of benzene with low hydrogen consumption under mild conditions, providing a new engineering solution for the conversion of lignin-derived phenolic compounds into high-value chemicals.

**Figure 3:**
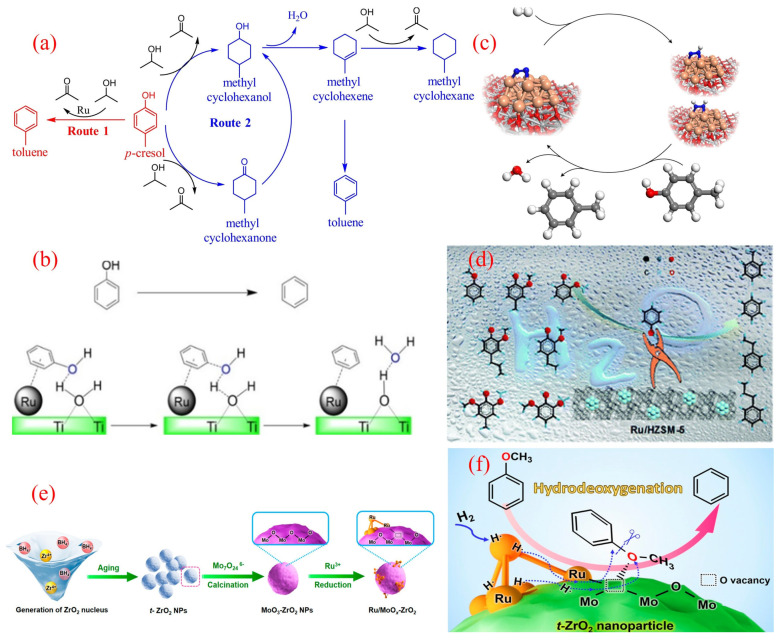
(**a**) Reaction pathway of p-cresol HDO over the Ru/Nb_2_O_5_-SiO_2_ catalyst [52]. Copyright 2017 Elsevier. (**b**) Water-assisted HDO of phenol over the Ru/TiO_2_ catalyst [53]. Copyright 2015 American Chemical Society. (**c**) The combination of N_2_ activation and an HDO reaction over the Ru/TiO_2_ catalyst [54]. Copyright 2019 Springer Nature. (**d**) The selective HDO of phenolics to BTX over Ru/HZSM-5 in water [55]. Copyright 2016 Royal Society of Chemistry. (**e**) Schematic synthetic process for MoO_x_-decorated ZrO_2_-supported Ru nanocluster catalysts; (**f**) The selective HDO of anisole to benzene over the Ru/MoO_x_-ZrO_2_ catalyst [56]. Copyright 2021 American Chemical Society.

The innovative application of hydrogen donor reagents provides an effective strategy for the directed conversion of phenolic compounds into BTX, replacing traditional hydrogen gas. Guo’s research team recently developed the Ru/Nb_2_O_5_-SiO_2_ catalytic system, which achieved the efficient conversion of p-cresol to toluene in a 2-propanol hydrogen-transfer medium [52]. The system achieved a toluene yield of 84.0% under reaction conditions at 230 °C, and its superiority lies in the significant reduction in the C-O bond dissociation energy by the NbO_x_ species and the synergistic effect of the moderate hydrogen-transfer activity of Ru nanoparticles. The analysis of reaction pathways (Figure 3a) shows that in the presence of 2-propanol, the system mainly follows the direct deoxygenation pathway (DDO, route 1), generating only trace amounts of methylcyclohexanone/alcohol intermediates (route 2, total selectivity of 5.2%), which can be further converted to toluene through subsequent dehydrogenation reactions. It is worth noting that compared to the molecular hydrogen system, the hydrogen-transfer strategy increases the selectivity of aromatic hydrocarbons, which is attributed to the steric hindrance effect of the donor alcohol effectively suppressing excessive hydrogenation of aromatic rings. This breakthrough work establishes a new paradigm for the preparation of BTX from lignin-derived phenols through a nonhydrogen atmosphere catalytic conversion, providing important theoretical support for the optimization of the hydrogen economy in biomass-refining processes.

The study of ruthenium-based catalytic systems has been further expanded to titanium dioxide supports, and the mechanism of Ru/TiO_2_ in the direct hydrogenation deoxygenation (HDO) of phenol has revealed a unique catalytic pathway mediated by water [53]. The initial benzene selectivity of the system was only 38% at 573 K and 3.79 MPa, while the introduction of 10 wt% water significantly increased the selectivity to 95%, attributed to the dynamic proton network formed by water molecules on the TiO_2_ surface. As shown in Figure 3b, the water molecules adsorbed on the surface of hydroxylated TiO_2_ achieve bidirectional proton transfer through the Ru/TiO_2_ interface. During the extraction of phenolic hydroxyl groups, the C-O bond dissociation energy is reduced through a proton synergistic transfer mechanism. The synchronous hydrogen overflow effect induces the generation of oxygen vacancies in TiO_2_, and its amphiphilic properties promote the cleavage of H_2_ to generate active hydrogen species, forming a continuous deoxygenation–dehydrogenation cycle. The latest research progress indicates that reactive atmosphere engineering can further optimize the performance of the system. The Duan team achieved the efficient conversion of cresol to toluene by introducing 6-bar N_2_ at 160 °C and 1-bar H_2_ (Figure 3c) [54]. Nitrogen forms hydrogenated nitrogen species on the Ru metal surface through chemical adsorption, and the activation energy of the Caryl-O bond is reduced through a proton-relay mechanism. At the same time, the nitrogen coating dynamically regulates the distribution of active sites, increasing the adsorption rate of cresol and reducing the apparent activation energy of the reaction. This gas–solid interface regulation strategy provides a new process enhancement approach for lignin catalytic conversion.

The research on ruthenium-based catalyst systems has further extended to the field of molecular sieve supports, and Ru/HZSM-5 has demonstrated excellent performance in the directional conversion of phenolic monomers (phenol, anisole, guaiacol, and eugenol) to benzene derivatives (BTX) [55]. In an aqueous reaction system composed of 240 °C, 2-bar H_2_, and 6-bar N_2_, the catalyst achieved 100% BTX selectivity for the conversion of guaiacol. In-depth research has shown that the topology of HZSM-5 has a decisive impact on catalytic performance: the cross-shaped HZSM-5-supported ruthenium catalyst (Ru/c-HZSM-5) achieved a 97% benzene yield under the same conditions. X-ray photoelectron spectroscopy (XPS) and infrared spectroscopy (IR) analysis revealed that the special pore structure of the cross-shaped molecular sieve induces the generation of electron-deficient ruthenium species, whose strong electronegativity significantly enhances the adsorption capacity of reactants. Additionally, the adsorption of guaiacol and hydrogen on cross-shaped Ru/HZSM-5 was more significant than other catalysts, which may be due to the abundant Lewis acid sites on cross-shaped Ru/HZSM-5, thus promoting the adsorption of oxygen atoms inside guaiacol (Figure 3d). The synergistic effect of electronic structure adsorption performance reduces the C-O bond dissociation energy, ultimately achieving the high-fidelity deoxygenation of aromatic ring structures [55].

The latest research breakthrough has achieved a performance leap of ruthenium-based catalysts in the aqueous hydrogenation deoxygenation (HDO) of benzyl ether through an interface engineering strategy [56]. The research team innovatively constructed a MoO_3_ modification layer on the surface of tetragonal zirconia (t-ZrO_2_) and formed a Ru^δ+^-Ov-Mo^5+^ ternary active interface by precisely loading Ru nanoclusters (Figure 3e). Under mild reaction conditions, the benzene selectivity of the catalyst reached 84.7%. Its performance leap is attributed to a unique electronic synergistic effect: MoO_x_ defect clusters induce Ru nanoclusters to exhibit partial oxidation states through charge transfer, significantly enhancing the chemical adsorption energy of methoxy groups (Figure 3f). Advanced microscopic and spectroscopic analyses revealed a precisely engineered architecture where atomic-scale Ru clusters coexist with defective MoO_x_ moieties on tetragonal zirconia (t-ZrO_2_) substrates. This unique configuration induces charge redistribution at metal–oxide interfaces, generating the electron-deficient Ru^δ+^ sites and coordinatively unsaturated oxygen vacancies. The established SMS creates robust electronic coupling between metallic Ru and MoO_x_ species. Such synergistic interactions not only optimize active-site configuration but also endow the hybrid system with exceptional thermal stability during prolonged catalytic operation [56].

#### 2.1.3. Platinum (Pt)-Based Catalysts

**Figure 4:**
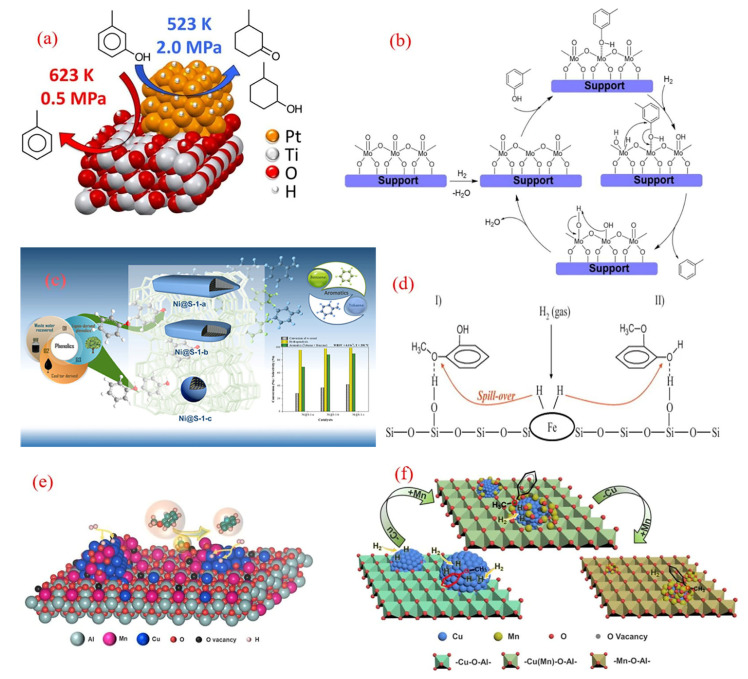
(**a**) The HDO reaction of m-cresol over the Pt/TiO_2_ catalyst [61]. Copyright 2016 American Chemical Society. (**b**) Mechanism of the direct deoxygenation route of m-cresol on a schematic molybdenum oxide-site species [62]. Copyright 2017 Elsevier. (**c**) The upgrading of phenolics to BTX over the different Ni@silicalite-1 catalysts [63]. Copyright 2021 American Chemical Society. (**d**) Possible reaction mechanism of the HDO conversion of guaiacol over the Fe/SiO_2_ catalyst [39]. Copyright 2012 Elsevier. (**e**) The HDO of anisole to benzene over the Mn-doped Cu/Al_2_O_3_ catalysts; (**f**) the mechanism for the anisole HDO on Cu/MnAlOx with the Mn/Cu molar ratio increasing [64]. Copyright 2021 Elsevier.

The platinum-based catalytic system exhibits unique advantages in the field of directed deoxygenation of phenolic compounds through precise interface regulation. The Pt/HBeta bifunctional catalyst developed by the Resasco team achieved the efficient conversion of meta cresol and benzyl ether under low-pressure conditions at 673 K [57,58]. The catalytic conversion frequency of this system was three times higher than that of a pure HBeta molecular sieve, and the BTX selectivity exceeded 90%. Mechanistic studies have shown that Pt nanoparticles and Brønsted acid sites form a dual-functional synergistic mechanism: the metal site selectively activates the adjacent aromatic ring of Caryl-OH to generate a hexadienol intermediate, and the adjacent acid site immediately triggers a dehydration reaction to complete the deoxygenation cycle (Figure 4a) [59]. Zhu’s research group further expanded the application of this system in the conversion of guaiacol and found that Pt/HBeta was regulated by a unique adsorption configuration—the aromatic ring plane adsorbed on the Pt crystal surface and the oxygen atom anchored acid site—resulting in a direct deoxygenation pathway contribution rate of over 85%, successfully inhibiting competitive reactions such as demethylation and decarbonylation [60].

A significant breakthrough has been made in the study of the structure–activity relationship between the carrier effect and reaction pathway of platinum-based catalysts. Griffin’s team systematically analyzed the performance differences between Pt/C and Pt/TiO_2_ in the hydrogenation deoxygenation (HDO) of meta cresol using an experimental computational strategy [61]. This study used a gas-phase flow reactor to simulate two process conditions, catalytic rapid pyrolysis (CFP: 623 K, 0.5 MPa) and hydroprocessing (HT: 523 K, 2.0 MPa), and conducted comparative analysis under a unified control of a 35% conversion rate. The experimental data show that under CFP conditions, the toluene carbon selectivity of Pt/TiO_2_ reaches 78%, which is significantly higher than that of Pt/C at 46%. Under HT conditions, both types of catalysts mainly generate 3-methylcyclohexanone/alcohol (selectivity >95%), which is highly consistent with Ruddy’s thermodynamic regulation law [65]: aromatic ring hydrogenation tends to be in the low-temperature region, while direct deoxygenation and acid-catalyzed reactions dominate the high-temperature region (>623 K). Mechanistic studies have shown (Figure 4a) that the Pt metal phase dominates the hydrogenation pathway at 523 K, while the oxygen vacancy effect of TiO_2_ support is significantly enhanced at 623 K, resulting in a decrease in deoxidation activation energy through metal–carrier electronic interaction. This temperature-dependent synergistic mechanism is reflected in the participation of TiO_2_ surface hydroxyl groups in proton-transfer networks at high temperatures, optimizing the Caryl-O bond dissociation pathway and increasing toluene selectivity.

Noble metal catalysts (e.g., Pd, Pt, Ru) exhibit unparalleled efficiency in phenolic HDO, primarily due to their exceptional hydrogen dissociation capabilities and tailored interactions with oxygen-containing functional groups. Despite their superior performance, noble metals face critical limitations, including high cost, susceptibility to coking during prolonged operation, and limited availability, which restrict their large-scale industrial adoption.

### 2.2. Non-Noble Metal Catalysts

Due to the high cost of noble metal-based catalysts, which makes them less viable for large-scale industrial applications, researchers have been developing cost-effective, non-precious metal-loaded catalysts as alternative systems for the conversion of phenolic compounds to benzene, toluene, and xylene (BTX). These systems have shown great promise in hydrodeoxygenation (HDO) processes.

The group of Prasomsri explored the use of a homogeneous MoO_3_ catalyst, under reaction conditions of 593 K, 3 h, and 0.1 MPa H_2_, and they achieved 94% overall conversion with high yields of benzene and toluene [66]. The success of the MoO_3_ catalyst was primarily attributed to its ability to selectively cleave the phenolic Ph-OMe bond over the weaker aliphatic Ph-O-Me bond. Advancing this research direction, Zhang’s team conducted a systematic evaluation of defect-engineered MoO_3_ catalysts for aromatic production under lower H_2_ environments [62]. Their work demonstrated effective phenol-to-benzene transformation (98.1% conversion, 99.5% selectivity) under mild hydrotreatment conditions (340 °C, H_2_/N_2_ = 1:6 *v*/*v* at 3.5 MPa total pressure). Mechanistic studies revealed that coordinatively unsaturated sites generated by surface oxygen vacancies served as active centers, facilitating selective C-O bond scission while inhibiting aromatic ring hydrogenation. Specifically, the oxygen vacancies (Mo^5+^ sites) acted as the active centers for phenol HDO, facilitating the adsorption of lone-pair electrons from phenol and enabling the hydrolysis of carbonyl intermediates to form benzene. Additionally, the introduction of N_2_ into the reactor prevented the over-reduction of MoO_3_, maintaining a high-oxygen-vacancy content on the catalyst surface and enhancing the HDO conversion of phenol to benzene.

The hydrodeoxygenation (HDO) of m-cresol to toluene was investigated using MoO_3_-based catalysts supported on various materials, including SiO_2_, Al_2_O_3_, TiO_2_, ZrO_2_, and CeO_2_ [67]. The catalytic systems selectively exhibited C-O bond scission capabilities while preserving aromatic unsaturation, enabling the effective transformation of m-cresol into toluene with notable conversion efficiency and exceptional selectivity. Supporting materials play a crucial role in molybdenum-based catalysts, especially in stabilizing specific low-oxidation-state molybdenum ions and affecting their reduction performance, with TiO_2_ and ZrO_2_ identified as the most effective supports for enhancing activity and stability. The reaction mechanism remained inconclusive, but the rate of reaction appeared to be influenced by ligand-unsaturated Mo sites, which are essential for the oxygen-vacancy-driven mechanism. Further studies by Vinicius et al. explored the impact of different supports on MoO_x_-based catalysts for m-cresol HDO at 340 °C and a total pressure of 4 MPa (Figure 4b) [68]. Regardless of the catalyst used, the selectivity for m-cresol to toluene consistently exceeded 80%, with the catalytic activity influenced by the nature of the support, following the order as follows: MoO_x_/Al_2_O_3_ > MoO_x_/SBA-15 > MoO_x_/SiO_2_. This variation was attributed to differences in the reducibility of Mo species, which could be enhanced by using acidic carriers like Al_2_O_3_ or mesoporous materials like SBA-15 compared to commercial SiO_2_. The proposed reaction mechanism involves the adsorption of oxygen from the reactant onto oxygen vacancies, followed by the heterolytic dissociation of H_2_ into proton and hydride species. The hydride adds to a carbon atom bearing an OH group, leading to the cleavage of the C-O bond and the formation of toluene. Oxygen vacancies are restored by releasing water during the process [67,69]. Mo species are present as polymolybdate phases, including Mo^6+^, which reacts with H_2_ to form MoO_x_ species (Mo IV) and release water, facilitating the breaking of C-O bonds [70]. This study highlights the importance of support materials in optimizing MoO_3_ catalysts for HDO processes, offering insights into the development of cost-effective and efficient catalytic systems for industrial applications [68].

However, despite these advantages, MoO_3_-based catalysts face challenges such as deactivation due to coke deposition and over-reduction, which can lead to the formation of inactive Mo species [62,68]. These limitations have prompted researchers to explore alternative non-precious metal catalysts for HDO processes.

Nickel-based catalysts have emerged as a promising alternative for the HDO of phenolics to BTX [71,72,73]. Researchers have investigated a wide range of Ni-containing catalysts supported on diverse carriers [74]. The comparative evaluation of nickel-based catalytic systems revealed significant carrier-dependent performance in aromatic production. When supported on microporous carbon matrices, Ni nanoparticles achieved an 80% benzene yield under optimized conditions (310 °C, 3-bar H_2_). The superior performance of the Ni/C catalyst was attributed to its strong acidic sites and excellent metal dispersion, which favored the selective cleavage of the C-O bond. Similarly, the Ni/TiO_2_ catalyst demonstrated high selectivity for benzene, albeit with limited anisole conversion, due to strong metal–support interactions [75]. These findings suggest that the catalytic activity and selectivity of Ni-based catalysts are significantly influenced by the nature of the support material.

The catalytic mechanism of Ni-based catalysts involves the adsorption of reactants onto ligand-unsaturated Ni sites, which promote the activation of hydrogen and stabilize transition states, leading to the cleavage of the C-O bond. The size of the Ni particles also plays a crucial role in determining reaction pathways and product selectivity [76]. Smaller Ni particles tend to enhance the conversion rate by providing more active sites, while larger particles may influence the selectivity toward specific products. This highlights the importance of optimizing the Ni particle size and support properties to achieve desired catalytic outcomes.

This study further explores the optimization of Ni-based catalysts for the hydrodeoxygenation (HDO) of phenolics to BTX. Notably, Ni/Ce_1−x_Nb_x_O_2_ catalysts were developed, demonstrating exceptional performance in the HDO of phenol. At 300 °C, the Ni/CeO_2_ catalyst achieved a remarkable 92% yield of benzene [77]. The reaction mechanism involves phenol tautomerization followed by hydrogenation to cyclohexanone and hydrolysis to methane [74]. The addition of Nb significantly alters the product distribution, with the increased Nb content enhancing benzene selectivity while reducing the formation of hydrogenation and hydrolysis products. This improvement is attributed to the niobium-rich surface and the strong interaction between phenol’s oxygen and the oxygenophilic Nb^5+^ sites, which promotes carbonyl hydrogenation.

In another approach, Ni@S-1 catalysts with varying crystal sizes were synthesized using an in situ encapsulation method and applied to m-cresol conversion (Figure 4c) [63]. These catalysts exhibited outstanding stability over 300 h, achieving 78.4% phenol conversion and a 73.1% BTX yield. The encapsulation of Ni nanoparticles within the zeolite matrix modulates reactant adsorption patterns and leverages the shape-selective properties of silicalite-1, enhancing aromatic selectivity and catalyst stability. The reduction in the Ni@silicalite-1 crystal size is further improved by selectively eliminating the hydroxyl groups that hindered the further hydrogenation of aromatics [63]. This design also prevents the migration and aggregation of Ni nanoparticles, maintaining catalyst performance even during long-term runs and avoiding undesired benzene ring reactions.

The exploration of cost-effective and environmentally friendly catalysts for the hydrodeoxygenation (HDO) of phenolics to BTX has recently expanded to include iron (Fe)- and manganese–copper (Mn-Cu)-based systems. In a notable study, Fe/SiO_2_ catalysts were investigated for the gas-phase HDO of guaiacol, achieving 74% conversion and a 38% BTX yield at 400 °C and 10 bar [39]. These Fe-based catalysts demonstrated advantages such as minimal aromatic ring hydrogenation and low-coking tendencies. The mechanism involves the adsorption of guaiacol’s oxygen atoms at weak acidic OH sites on the silica surface, favoring C-O bond cleavage over C-C bond breakage [64]. Active hydrogen species, derived from H_2_ dissociation on Fe particles, play a critical role in facilitating the reaction (Figure 4d). This work highlights Fe as a promising, sustainable catalyst for lignin-to-BTX production via rapid pyrolysis and the subsequent HDO of lignin vapors.

In another development, Mn-doped Cu/Al_2_O_3_ catalysts, synthesized from layered double hydroxides, were tested for the liquid-phase HDO of anisole (Figure 4e) [78]. The addition of Mn significantly enhanced benzene selectivity, reaching ~65% for the 4Cu/8Mn_4_AlO_x_ catalyst—six times higher than that of undoped Cu/Al_2_O_3_. Structural studies revealed that MnO_x_ doping optimized the surface structure of Cu particles and generated a high density of oxygen vacancies (OVs). These OV sites, along with metallic Cu, synergistically activated the C-O bond in anisole, improving HDO activity. However, excessive Mn/Cu ratios led to MnO_x_ encapsulating Cu particles, reducing the active sites for H_2_ activation and suppressing catalytic performance. This study demonstrates the potential of Mn-Cu/Al_2_O_3_ catalysts for selective and efficient HDO processes.

Non-noble metal catalysts (e.g., Mo, Ni, Fe, Mn-Cu) offer cost-effective alternatives for HDO, leveraging earth-abundant elements to achieve selective deoxygenation. However, these catalysts often suffer from lower intrinsic activity compared to noble metals, as well as challenges like metal leaching (Ni) and redox instability (Mo), requiring further advancements in stability and regeneration protocols.

### 2.3. Bimetallic Catalysts

The addition of a second metal can modify the electronic or structural properties of the primary-metal-loaded catalyst compared to its monometallic counterpart, potentially enhancing catalytic activity, selectivity, and durability. This modification can occur through changes in the d-band electron density of the primary metal or by altering its surface configuration [34,79,80,81,82]. Additionally, the two metals may fulfill distinct roles during the reaction process [83].

#### 2.3.1. Fe-Containing Bimetallic Catalysts

**Figure 5:**
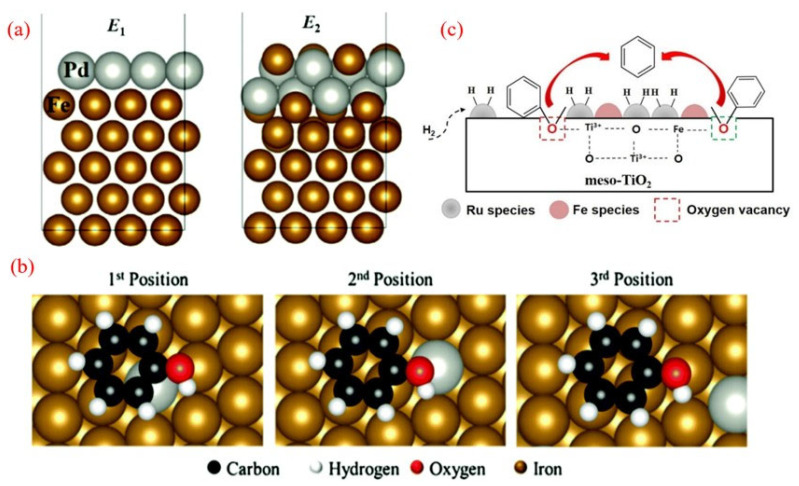
(**a**) The structure of the Pd layer in the matrix on the Fe (110) surface. (**b**) Adsorption conformation of phenol on the Fe (110) surface Pd atom. The distance from the adsorbate to the palladium atom increases from position 1 to position 3 [84]. Copyright 2013 Elsevier. (**c**) The possible mechanism of the anisole HDO over the RuFe/meso-TiO_2_ catalyst [86]. Copyright 2018 Elsevier.

The oxophilic nature of iron (Fe) makes it highly effective for deoxygenation processes, particularly due to its strong affinity for oxygen-containing groups in phenolic compounds. However, Fe has a limitation: its poor ability to dissociate H_2_ [34]. To overcome this, researchers developed a carbon-supported PdFe bimetallic catalyst, which enhances the overall hydrodeoxygenation (HDO) rate while maintaining efficient oxygen removal during the HDO of guaiacol [84]. This PdFe/C catalyst demonstrated superior performance, achieving an 83.2% benzene yield at 450 °C, significantly outperforming the monometallic Fe/C catalyst, which yielded only 43.3%. The possible structures of the bimetallic catalyst, as shown in Figure 5a, include a monolayer of Pd on the Fe (110) surface or a mixed surface of Pd and Fe atoms. Characterization and density functional theory (DFT) studies reveal that the Pd-Fe alloy surface is enriched with Pd nanodots, while Fe particles dominate the subsurface, indicating that Fe serves as the primary active site for the reaction. Meanwhile, Pd modifies the electronic structure, enhancing catalytic activity. Figure 5b revealed three distinct adsorption configurations on the catalyst surface. Adsorption energetics analysis demonstrated a positive correlation between the binding strength and proximity to Pd active centers, with the third adsorption position exhibiting the most thermodynamically favorable adsorption energy, conclusively establishing preferential phenol adsorption at the Fe surface sites. As phenol interacts strongly with the Fe surface, the C-O bond weakens, making the HDO of phenol more susceptible.

The addition of a second metal can create additional active sites, often acidic in nature, which can significantly enhance deoxygenation [34,85]. Phan et al. demonstrated that incorporating Fe into a Ru/meso-TiO_2_ catalyst drastically alters its surface properties and catalytic performance, achieving over 80% benzene selectivity in the HDO of anisole at 250 °C and 1 MPa of H_2_ [86]. In this system, Ru particles efficiently dissociate H_2_, while the oxygenophilic Fe sites strengthen interactions between the oxygen-containing functional groups and the carrier surface. The synergistic interaction between Ru and Fe enhances C-O bond cleavage without ring hydrogenation, thereby minimizing H_2_ consumption (Figure 5c). Additionally, the bimetallic catalyst’s improved activity is linked to an increase in oxygen vacancies on the carrier surface. Similarly, a bimetallic FeReO_x_/ZrO_2_ catalyst was investigated for the mild HDO of various phenolic compounds [26]. Notably, at 250 °C, it delivered a BTX yield of 50.5% in the HDO of m-cresol, along with high stability, retaining 93.7% activity after 160 consecutive reactions. The catalyst’s mesoporous structure, oxygenophilic nature, and balanced acidity—induced by rhenium oxide and zirconium oxide carriers—contribute to its high dehydration efficiency. This system offers a cost-effective, sustainable route for BTX production under atmospheric pressure at 350 °C, bringing functional chemicals from lignin biorefining closer to commercialization.

**Figure 6:**
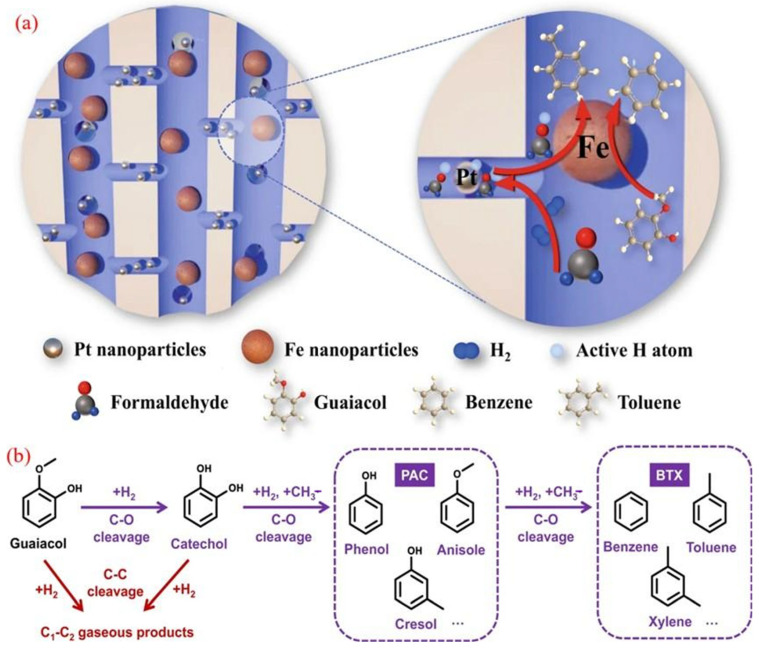
(**a**) Hydrogen spillover enhanced by oxygenate additives during catalysis; (**b**) the suggested HDO route of guaiacol to produce BTX [88]. Copyright 2022 Springer Nature.

The key role of the hydrogen overflow effect in the heterogeneous catalytic hydrogenation deoxygenation (HDO) process has been experimentally verified [87]. Taking the bimetallic Pt@Fe@SiO_2_ system as an example [88], the catalyst accurately constructs a hierarchical pore structure through a dual-mode plate method: Pt nanoparticles are located in SiO_2_ micropores, and Fe nanoparticles are distributed in macropores. In the HDO reaction of guaiacol, although it is difficult to achieve surface hydrogen migration with SiO_2_ as a non-reducible carrier, the introduction of C_1_ to C_3_ carbonyl compounds or ester oxygen-containing additives allows the active hydrogen atoms generated at Pt sites to migrate across scales to Fe active sites, improving the migration efficiency (Figure 6a). This molecular-mediated hydrogen-transfer mechanism significantly enhances the deoxygenation activity of bimetallic systems compared to single-metal Pt/Fe catalysts.

Dynamics analysis shows that oxygen-containing additives reduce the activation energy of the key hydrogenation steps on the Fe surface by stabilizing transition-state hydrogen species. It is worth noting that such hydrogen carriers can be generated through the in situ dehydrogenation or steam reforming of alcohol precursors, simultaneously providing H_2_ sources for BTX synthesis. This study has pioneered a new strategy of “molecular assisted hydrogen overflow”, providing a universal solution for the synergistic catalysis of spatially isolated active sites on inert carriers.

#### 2.3.2. Ni-Containing Bimetallic Catalysts

Ni-based catalysts possess superior hydrogenation abilities for biomass upgrading. However, their limited propensity for oxygen removal restricts their application in HDO reactions [89,90]. As a result, numerous bimetallic Ni catalysts have been developed to enhance their deoxygenation efficiency. Tao and colleagues developed highly reactive NiMo bimetallic catalysts [91]. Interestingly, the synthesized Ni-Mo/SiO_2_ catalyst demonstrated remarkable selectivity (>96%) for BTX and achieved a 99% conversion rate under low-H_2_ partial-pressure conditions. Additionally, the catalyst’s plentiful acid sites significantly promoted the methyl-transfer reaction, leading to reduced carbon losses. Similarly, Yang et al. reported that the bimetallic Ni-Mo/SiO_2_ catalyst exhibited high activity and selectivity (>80%) for the HDO of m-cresol to toluene across a broad temperature range (250–350 °C) under atmospheric hydrogen pressure. Further confirmation revealed that the interaction between the Ni core surface and the optimal MoO_x_ species enhancing the Ni surface is crucial for attaining high activity and selectivity [92]. This study offers novel insights into cost-effective HDO processes for products derived from lignin thermal degradation.

**Figure 7:**
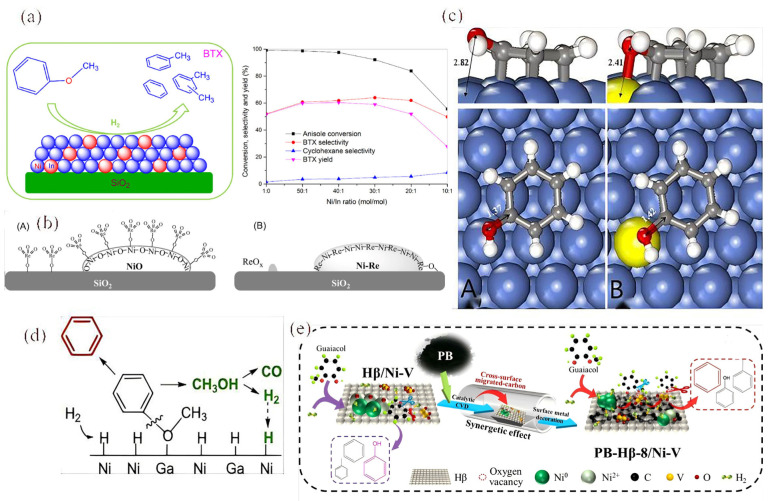
(**a**) The performance of the In on Ni/SiO_2_ catalyst in the HDO of anisole with different products [93]. Copyright 2017 Elsevier. (**b**) Schematic representation of the structure of the bimetallic Ni-Re catalyst before (**left**) and after (**right**) reduction at 450 °C [95]. Copyright 2017 Elsevier. (**c**) Phenol adsorption onto the Ni (**A**) surface and (**B**) phenol adsorption onto the (Re)Ni (111) surface [95]. Copyright 2017 Elsevier. (**d**) The plausible reaction mechanism in the HDO of anisole on Ni_x_Ga/SiO_2_ [96]. Copyright 2017 Elsevier. (**e**) The proposed mechanism of modification between aromatic selectivity, oxygen vacancies, and H_2_ absorption [97]. Copyright 2021 American Chemical Society.

Wang’s group studied the influence of indium on the catalytic performance of Ni/SiO_2_ in the hydrodeoxygenation (HDO) [93]. Under conditions of 300 °C, 0.1 MPa, and a H_2_/anisole ratio of 25, the Ni_40_In/SiO_2_ catalyst with 40% Ni content achieved a higher BTX yield of 60.4%, compared to that of 51.6% from the Ni/SiO_2_ catalyst (Figure 7a). They found that the size of the Ni-In bimetallic microcrystals was similar to that of monometallic Ni with the same Ni content. However, the bimetallic Ni-In catalyst absorbed significantly less H_2_ because the In atoms diluted the Ni atoms.

Charge transfer from In to Ni was observed in the bimetallic Ni-In catalysts, indicating a close interaction between the Ni and In atoms, along with geometrical and electronic modifications of Ni by In. In anisole HDO, the Ni-In bimetallic catalysts showed lower activity than monometallic Ni but exhibited higher selectivity for BTX. Furthermore, the bimetallic catalysts demonstrated reduced methanation activity, leading to higher carbon yields and decreased hydrogen consumption [94]. Additionally, a lower Ni/In ratio resulted in a greater impact of In on catalytic performance, with BTX selectivity primarily determined by the Ni/In ratio and nearly independent of the Ni content [93].

Furthermore, the geometric and electronic effects of bimetallic Ni-Re catalysts for the selective HDO of m-cresol to toluene were further investigated [95]. The incorporation of Re into the Ni/SiO_2_ catalyst stabilizes highly dispersed NiO through robust Ni-O-Re interactions. As anticipated, the bimetallic catalysts exhibit both geometric and electronic effects: (1) Re disperses the Ni surface into smaller aggregates and creates Ni-Re adjacent sites, and (2) the proximity between Ni and Re reduces the occupancy of the Ni d-band. Unlike the adsorption of phenol on the bare Ni (111) surface via the benzene ring (with O pointing away from the surface), the adsorption of phenol on the (Re)Ni (111) surface occurs via the benzene ring on Ni and the O atom on Re at Ni-Re adjacent sites, which facilitates C-O bond cleavage (Figure 7c). The incorporation of Re in the surface alloy modifies the Ni surface by breaking it into smaller, discrete regions (geometric effect) and reduces the d-band electron density of Ni (electronic effect) [95]. DFT calculations indicate that the Ni-Re neighboring site facilitates the cleavage of the C-O bond by adsorbing the O atom on Re and the benzene ring on the adjacent Ni atom, thus enhancing deoxygenation and promoting toluene production. At the same time, the reduced electron density in the Ni d-band weakens the adsorption of benzene rings on the surface, preventing the C-C hydrogenolysis of aromatic products.

The bimetallic Ni_x_Ga/SiO_2_ catalysts were synthesized using the impregnation method followed by reduction at 550 °C and were evaluated for the vapor hydrodeoxygenation (HDO) of anisole at 0.1 MPa and 300 °C [96]. The results demonstrated that Ni_x_Ga/SiO_2_ exhibited higher anisole conversion and benzene selectivity compared to the monometallic Ni/SiO_2_, while also showing lower selectivity for phenol. Specifically, at the same anisole conversion rate of 31%, the selectivities for benzene were 75.2%, 83.0%, and 92.6% for Ni/SiO_2_, Ni_6_Ga/SiO_2_, and Ni_3_Ga/SiO_2_, respectively.

The preferential cleavage of the CAr-O bond on the Ni-Ga alloy and the Ni_3_Ga intermetallic compound (IMC) results from the synergistic effects between adjacent Ni and Ga sites, as illustrated in Figure 7d. In the case of the Ni-Ga alloy and Ni_3_Ga IMC, the oxygen in anisole is more likely to be adsorbed at Ga sites, where Ga shows a stronger affinity for O compared to Ni, as inferred from H_2_ studies. This sequence of interactions ultimately leads to the cleavage of the CAr-O bond, allowing for the formation of benzene. In summary, the Ni_x_Ga/SiO_2_ bimetallic catalyst achieved a higher benzene yield while significantly lowering hydrogen consumption, marking a crucial advancement for the rational design of future catalysts in biomass upgrading applications.

In an innovative approach, biochar-modified Hβ/Ni-V catalysts were developed and tested for the atmospheric hydrodeoxygenation (HDO) of guaiacol to produce benzene, toluene, and xylene (BTX) [97]. Among these, the catalyst modified with pine nut shell biochar (PB), specifically PB-Hβ-8/Ni-V, achieved the highest selectivity for benzene and toluene at 69.17%. This enhancement in selectivity was attributed to the synergistic effects between the biochar and the Hβ/Ni-V catalyst. The mechanism behind this improvement involves the formation of a stable carbon layer on the Hβ/Ni-V catalyst, created through a metal-catalyzed chemical vapor deposition process utilizing volatiles from pyrolyzed PB (as illustrated in Figure 7e). In addition to forming this carbon layer, the thermal reduction of the carbon effectively decorated the surface metal, increasing the availability of active sites, particularly Ni^0^ and V^3+^. This increase in Ni^0^ sites enhances the catalyst’s ability to adsorb and dissociate hydrogen, thereby boosting its hydrogenation activity.

Moreover, the presence of biochar enhances the affinity of the catalyst surface for reactants, along with creating more oxygen vacancies [98]. This combination contributes to the improved selective adsorption of oxygen-containing groups and facilitates the breaking of CAr-OH bonds, leading to enhanced deoxygenation activity [97]. Overall, the incorporation of biochar modifications significantly improved the HDO activity of the catalysts, resulting in a greater yield of BTX. The use of pyrolytic biochar also presents a promising and cost-effective alternative to traditional zeolite–metal catalysts, making it a viable option for sustainable biomass conversion strategies.

#### 2.3.3. Re- and W-Based Bimetallic Catalysts

In addition to Ni- and Fe-based bimetallic catalysts, a variety of other bimetallic combinations, including Pd-Re, Re-Mo, Re-V, Ru-W, and Pt-Wu, have been developed and utilized for converting various phenolic compounds into benzene, toluene, and xylene (BTX) [99,100,101,102,103,104]. For instance, research conducted by Pouya et al. found that ZrO_2_ showed better catalytic performance compared to cerium dioxide, attributed to its higher acid density, which enhances catalytic activity. All the hydrogenated metals were effective in promoting the hydrogenation of carbonyl groups, leading to selective BTX production. Among the developed catalysts, PdReO_x_/ZrO_2_ exhibited the highest BTX yield of 77.2%. This high activity is partially due to the weak acid strength contributed by the zirconium oxide carrier and rhenium oxide, which prevents phenolic adsorption and trapping at lower temperatures [99].

In another study focusing on anisole HDO, bimetallic Re-MoO_x_/TiO_2_ and Re-VO_x_/TiO_2_ catalysts were evaluated under conditions of 300 °C and 3 MPa of H_2_ [101]. Re-based catalysts exhibited a unique ability to favor the production of aromatic compounds due to their oxygenophilic properties. When combined with Mo and V cations in lower oxidation states, the selectivity for desired aromatic products like benzene and toluene was significantly improved. This enhanced selectivity can be attributed to the stronger adsorption of anisole on the surface oxygen vacancy sites of MoO_x_ or VO_x_. However, the extent of this improvement depended on the specific combination of Re with Mo or V, highlighting the synergistic interactions present between the Re and MoO_x_ species in particular. The catalytic activity, measured in terms of the intrinsic reaction rate, was determined by the nature of the specific surface species present. For instance, in the Re-MoO_x_/TiO_2_ catalyst, the activity was primarily linked to the exposed Mo^5+^ sites, while in the Re-VO_x_/TiO_2_ catalyst, the activity was mainly influenced by the Re^4+^ sites [101]. This research underscores the critical importance of designing catalysts with specific active sites tailored for the formation of targeted products, facilitating more efficient pathways for the transformation of lignin-derived phenolic compounds into valuable aromatic products like BTX.

**Figure 8:**
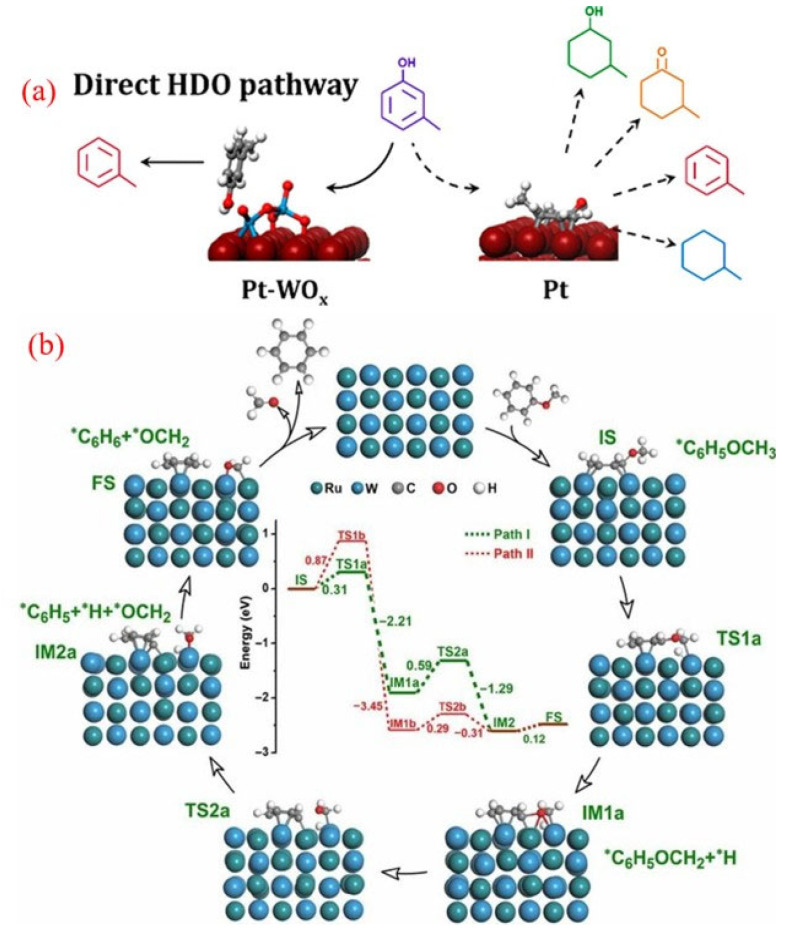
(**a**) The direct HDO of m-cresol over WO_x_-decorated Pt/C catalysts [103]. Copyright 2018 American Chemical Society. (**b**) The tentative reaction mechanism for the SSH reaction of anisole on a RuW/SiO_2_ catalyst [104]. Copyright 2019 Springer science.

Bimetallic tungsten (W)-based catalysts have shown remarkable catalytic performance for hydrodeoxygenation (HDO). Notably, the Pt-WO_x_/C catalyst exhibited exceptional activity and selectivity for the HDO of m-cresol (Figure 8a) [103]. Under various reaction conditions, this catalyst achieved over 94% selectivity for the desired products and demonstrated high stability, showing resistance to deactivation from coking. In contrast, the Pt/C catalyst showed lower selectivity for toluene production during the HDO process of m-cresol.

Reactivity studies, along with density functional theory (DFT) calculations, revealed that the HDO reaction on the Pt-WO_x_/C catalyst occurs via the direct hydrogenolysis of the C-O bond in m-cresol, which is adsorbed onto the oxygen vacancy sites present on the WO_x_ species. The interaction between Pt and the WO_x_ complexes not only stabilizes the oxide but also reduces the energy barrier for the formation of oxygen vacancies. These vacancies are crucial for creating redox sites on WO_x_ that actively participate in the direct C-O bond hydrogenolysis of adsorbed phenolics [103]. Moreover, the selective adsorption at these sites reduces the interaction of the aromatic ring with the Pt surface, thus minimizing the occurrence of side reactions.

Additionally, pioneering work by Meng and colleagues recently introduced a novel self-supported hydrogenolysis (SSH) process for converting aromatic ethers to BTX, utilizing hydrogen generated from the reactants themselves [104]. The RuW alloy nanoparticles served as efficient catalysts for these reactions. Notably, this method is unique because it eliminates the need for external hydrogen or other reducing agents and avoids the hydrogenation of the aromatic ring.

Mechanistic studies suggest that the adjacent Ru and W species in the RuW alloy nanoparticles work synergistically to enhance the SSH reaction. DFT studies (Figure 8b) identified two primary reaction pathways for the SSH of anisole with benzene: In path I, the aliphatic C-H (C_Al_-H) bond is first activated to produce a *C_6_H_5_OCH_2_ + *H intermediate (IM1a), followed by the hydrogenolysis of the C_Ar_-O bond in *C_6_H_5_OCH_2_ to generate benzene and formaldehyde (path I, green dashed lines). Path II involves initial cleavage of the C_Ar_-O bond to a *C_6_H_5_ + *OCH_3_ intermediate (IM1b), followed by the activation and dissociation of the C_Al_-H bond in *OCH_3_ to formaldehyde and benzene (Path II, red dashed line). This innovative, hydrogen-free, and efficient approach presents significant potential for producing functional chemicals from renewable biomass resources. The discovery of this method has opened avenues for exploring numerous other reactions and optimizing biomass conversion technologies.

Bimetallic catalysts enhance HDO performance through synergistic interactions between two metals, optimizing active-site configuration and reaction pathways. These systems balance activity, selectivity, and stability while reducing noble metal loading, but their complex synthesis and thermal management requirements pose challenges for industrial scaling.

### 2.4. Transition Metal-Based Catalysts

#### 2.4.1. Metal Sulfide Catalysts

Transition metal sulfides represent classic catalysts widely researched for hydrogenation reactions, demonstrating superior activity compared to transition metal oxides. They are extensively utilized in the hydrogenation of petroleum and coal-based liquid fuels, offering seamless compatibility with large-scale industrial hydrogenation units and catalyst production facilities [2,105,106,107].

The cobalt molybdenum sulfur (CoMoS) catalyst system has made significant progress in expanding its application from traditional petroleum refining and hydrogenation desulfurization (HDS) to biomass catalytic deoxygenation [108,109]. The Bui team conducted the first systematic study on the carrier effect of a CoMoS catalyst in the guaiacol HDO reaction [110] and found that compared with the commonly used Al_2_O_3_ carrier in industry, the CoMoS system loaded with ZrO_2_ increased the benzene selectivity to 42%. Its superiority lies in the strong electronic interaction between the CoMoS active phase and the carrier. However, the CoMoS catalyst obtained by traditional preparation methods has bottleneck problems such as high-temperature sulfur loss and rapid deactivation [111].

In response to the above challenges, the Liu team innovatively developed a novel catalyst that anchors isolated Co atoms with single-layer MoS_2_ [111]. By using H_2_ high-temperature treatment to construct sulfur vacancy defects on the MoS_2_ substrate, Co atoms are covalently bonded with vacancies to form Co-S-Mo active sites, exhibiting excellent activity, selectivity, and stability without sulfur loss in the 4-methylphenol conversion reaction. This breakthrough provides a new paradigm for the design of highly stable sulfide catalysts for biomass HDO processes.

**Figure 9:**
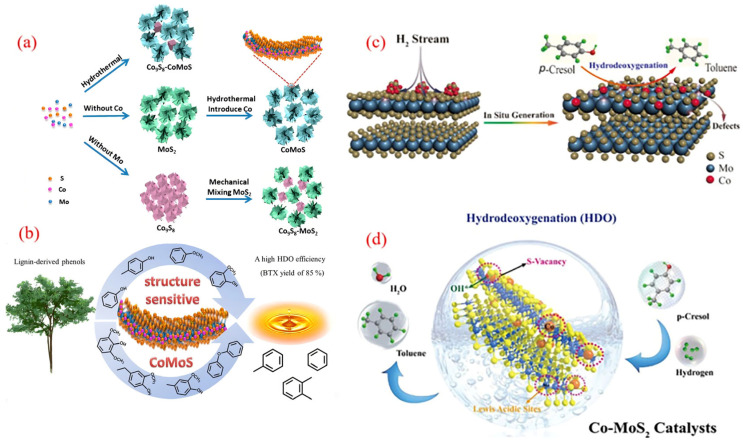
(**a**) Surface engineering of the CoMoS catalyst for the conversion of the phenolic HDO to the product BTX; (**b**) synthesis diagram of cobalt-doped MoS_2_ nanohybrids [112]. Copyright 2019 American Chemical Society. (**c**) Illustration of the hydrogen-flow-induced in situ formation of active CoMoS sites for the selective HDO of p-cresol to toluene [104]. Copyright 2020 American Chemical Society. (**d**) Tailoring of surface acidic sites in Co-MoS_2_ catalysts for the HDO of p-cresol to toluene [114]. Copyright 2021 American Chemical Society.

Song et al. demonstrated a surface atom engineering strategy that significantly enhanced the catalytic activity and selectivity of sulfide catalysts by achieving well-dispersed Co-doped MoS_2_ nanomaterials, maximizing the Co-Mo-S phase for efficient phenolic conversion to BTX (Figure 9a) [112]. Their self-induced method optimally modulates Co-substituted S sites, allowing Co atoms to bond well to the upper surface of the MoS_2_ nanosheets while preserving their structure. The characterization results showed a high density of Co-Mo-S phases on the catalyst’s surface, contributing to accelerated HDO reactions. As a result, using the engineered CoMoS catalyst led to the effective conversion of mixed phenols to BTX, achieving high yields exceeding 85% (Figure 9b). These findings may inspire further surface engineering of transition metal-doped catalytic nanomaterials.

Liu’s group utilized zeolitic imidazolate framework-67 as a cobalt precursor and template, employing a simple hydrothermal method to create CoMoS catalysts by depositing MoS_2_ ultrathin sheets inside the framework [113]. The optimized CoMoS-0.18 catalyst achieved a 92.4% conversion of cresol and a 95.5% selectivity for toluene at 250 °C. This high performance was linked to the development of accessible surface CoMoS phases. Additionally, H_2_ preactivation facilitated the creation of sulfur vacancies in MoS_2_, enhancing CoMoS interfaces through surface recombination. As illustrated in Figure 9c, the in situ generation of the CoMoS phase highlights the importance of H_2_ preactivation in boosting HDO activity. Both showed improved p-cresol conversion and toluene selectivity after H_2_ pre-reduction, suggesting a significant enhancement of the HDO pathway’s activity.

In another approach, Zhang and colleagues introduced a straightforward H_2_O_2_ etching method to modify the concentration of acidic sites on the CoMoS catalyst surface (Figure 9d) [114]. By adjusting the stoichiometric ratio of H_2_O_2_ to MoS_2_, the researchers optimized sulfur defects on the MoS_2_ surface, thereby stabilizing cobalt species. This process culminated in the formation of active CoMoS catalytic sites. The optimized Co-MoS_2_-2 catalyst exhibited the highest density of acidic sites, which translated into a 3.4-fold enhancement in catalytic activity for the hydrodeoxygenation (HDO) of toluene derived from p-cresol, compared to the baseline Co-MoS_2_ sample. Furthermore, a direct correlation was observed between the HDO activity and the surface acid content (encompassing both Lewis and Brønsted acidity) within the Co-MoS_2_ catalysts. This relationship is crucial for effectively cleaving C-O bonds, highlighting the importance of surface acidity in determining catalytic performance. Overall, enhancing surface acidic sites represents a promising strategy for designing more efficient catalytic systems.

**Figure 10:**
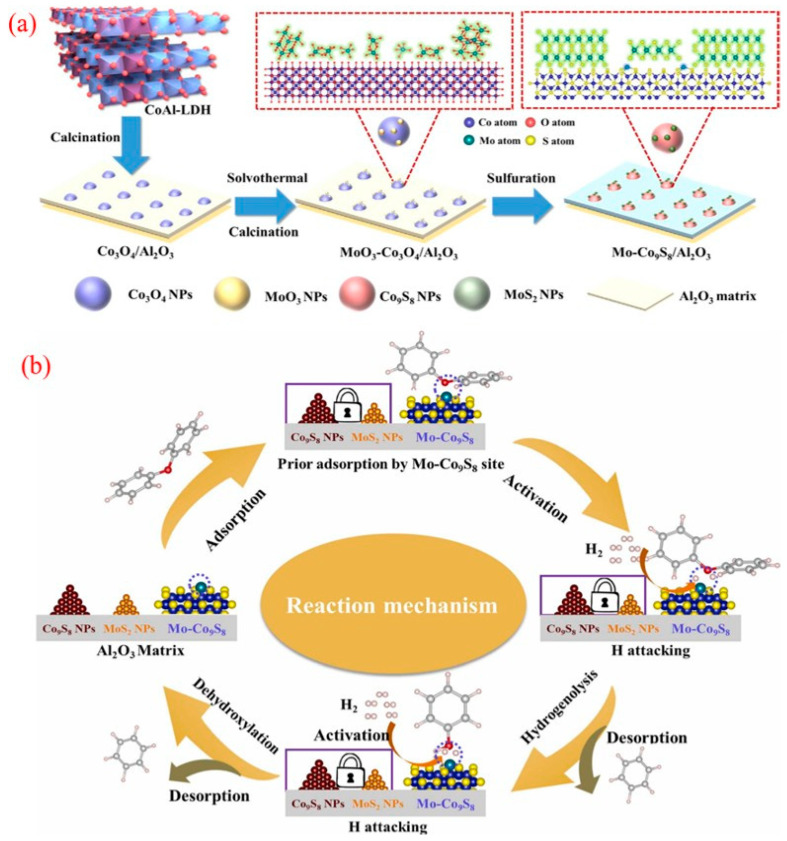
(**a**) Schematic illustration of the preparation of the Mo-Co_9_S_8_/Al_2_O_3_ catalyst; (**b**) illustration on the catalytic mechanism of the DPE HDO reaction over the eMo-Co_9_S_8_ site [115]. Copyright 2022 Elsevier.

A robust catalyst composed of Mo-doped Co_9_S_8_ nanoparticles supported on an Al_2_O_3_ matrix was successfully developed through a simple method using CoAl-hydrotalcite as a precursor. This multifunctional Mo-Co_9_S_8_/Al_2_O_3_ catalyst demonstrates exceptional hydrodeoxygenation (HDO) activity and selectivity in converting lignin to benzene, as illustrated in Figure 10a [115]. The catalyst facilitates the electron transfer from the Co to Mo sites within the Mo-Co_9_S_8_ structure, enabling the effective adsorption of oxidized compounds and contributing to its outstanding HDO performance. Notably, during the HDO of diphenyl ethers (DPEs), the catalyst achieved an impressive 99.8% conversion and a 91% yield of benzene, maintaining stability over at least 10 reaction cycles at 265 °C. The Mo-Co_9_S_8_ structure forms the covalent Mo-S-Co bonds on the Co_9_S_8_ surface, enhancing its ability to adsorb and activate oxygen-containing substrates. This feature promotes the efficient cleavage of C-O bonds while minimizing the unwanted hydrogenation of benzene rings, further highlighting its catalytic efficiency and selectivity.

The proposed catalytic mechanism for HDO over Mo-Co_9_S_8_/Al_2_O_3_, as depicted in Figure 10b, involves several critical steps. Initially, the negatively charged carbonyl oxygen in DPEs is adsorbed by the unsaturated Mo sites on the catalyst surface, which weakens the C-O bond. The electron transfer from Co to Mo enhances hydrogen dissociation activity, enabling dissociated hydrogen species at the Mo-Co_9_S_8_ site to attack the C-O group. This results in the effective cleavage of the 4-O-5 bond, ultimately producing phenol and benzene [116]. Subsequently, phenol undergoes a similar adsorption and activation process to convert into benzene through deoxygenation. This strategy highlights an innovative approach for rationally designing efficient and stable sulfide catalysts suitable for high-temperature reactions.

In addition to cobalt, nickel (Ni) serves as another promoter in sulfide catalysts primarily to enhance activity, although it often negatively affects aromatic selectivity [117,118]. Wang and colleagues explored the effectiveness of NiMoS_2_ catalysts in the hydrodeoxygenation (HDO) of cresol and found that adding Ni increases the catalyst’s activity [106]. However, they observed a decline in the selectivity for toluene as the Ni content increased. To further investigate, the authors synthesized NiS_x_ using a microwave-assisted hydrothermal method, which was then mixed with MoS_2_ to promote cresol HDO at 300 °C and 40 bar [119]. The results indicated that the combination of NiS_x_ with MoS_2_ improved conversion rates but did not alter product distribution. Comparisons between Ni-Mo-S, NiS_x_/MoS_2_, and physically mixed NiS_x_ + MoS_2_ suggested that synergies between NiS_x_ and MoS_2_—beyond merely forming a Ni-Mo-S phase—were key to enhancing activity [106].

The researchers proposed a remote-control model to explain their findings: cresols predominantly adsorb on MoS_2_ sites and are subsequently transferred to MoS_2_, where they react with hydrogen atoms that are dissociated on NiS_x_ sites. This interaction highlights the importance of using NiS_x_ in conjunction with MoS_2_ to optimize performance in cresol HDO reactions, balancing high activity with product selectivity [107,109,120].

#### 2.4.2. Transition Metal Phosphides

Transition metal phosphides, such as molybdenum phosphide (MoP), have emerged as highly effective catalysts for the hydrodeoxygenation (HDO) of phenolic compounds. MoP has demonstrated superior performance compared to its sulfide and oxide counterparts, exhibiting higher catalytic activity, lower activation energy, and greater selectivity towards aromatic compounds in the HDO conversion of p-cresols [121]. The enhanced catalytic performance of MoP is primarily attributed to the increased electron density of molybdenum in its phosphide form. This higher electron density around the molybdenum center likely enhances its nucleophilic properties, which can improve the catalyst’s ability to bind reactants and facilitate bond cleavage. Consequently, this leads to faster reaction kinetics and a lower activation energy, making the catalytic process more efficient.

According to molecular orbital theory, the lowest unoccupied molecular orbital (LUMO) of the C-O bond is antibonding, meaning that electrons donated from the catalyst to this orbital can facilitate C-O bond dissociation. Consequently, MoP, possessing the highest electron density among the catalysts studied, shows the strongest activity [122]. In experiments, MoP achieved a toluene selectivity of 60% and a conversion of 58% at 623 K and 4.4 MPa. To improve the selectivity for benzene in BTX production, Rensel and colleagues incorporated oxophilic iron (Fe) into the MoP catalyst, resulting in the bimetallic FeMoP catalyst. This bimetallic formulation produced benzene with up to 90% selectivity during the conversion of phenol at 673 K and 2.1 MPa of hydrogen [123]. Similarly, the conversion of anisole using the FeMoP catalyst also reached 90% selectivity for benzene. Further investigations of the FeMoP catalysts revealed the presence of two distinct metal sites: the metal site itself and a coordination unsaturation site (CUS) [124]. The CUS site likely arises from Mo^δ+^ species on the catalyst surface and is proposed to act as the active site for binding the hydroxyl group in phenol, destabilizing the Caryl-O bond. This bond is subsequently cleaved by hydrogen dissociated from the metal site, enhancing the overall HDO activity and selectivity of the catalyst [125].

A series of phosphide catalysts, including MoP, Fe_2_P, Co_2_P, WP, and Ni_2_P, have been utilized for the hydrodeoxygenation (HDO) of phenolics into BTX [126,127,128]. Among these, Ni_2_P-based catalysts demonstrated superior conversion of phenolics and higher selectivity for BTX, attributed to their desirable stability and deoxygenation capabilities [34]. The Ni_2_P/SiO_2_ catalyst achieved the highest turnover frequency and a benzene selectivity of 60%. Kinetic studies revealed an apparent activation energy of 40 kJ/mol for Ni_2_P/SiO_2_, lower than the activation energy for direct C-O bond cleavage. This suggests that increased benzene production is primarily due to the hydrogenation of the C=C double bond in the aromatic ring, followed by dehydration.

**Figure 11:**
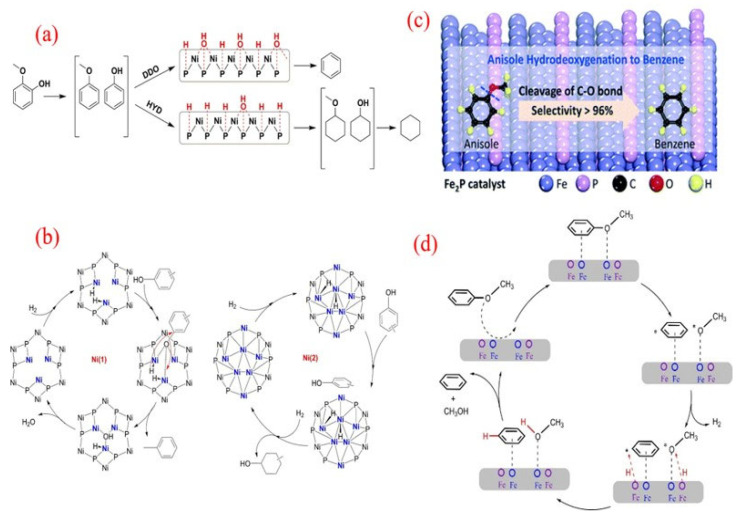
(**a**) The proposed reaction routes of the HDO of guaiacol over the Ni_2_P catalyst [129]. Copyright 2014 Elsevier. (**b**) Different Ni sites on Ni_2_P for the deoxygenation of cresol [132]. Copyright 2017 Elsevier. (**c**) The HDO of anisole to benzene over the Fe_2_P catalyst; (**d**) the possible reaction route of anisole HDO over Fe_2_P [135]. Copyright 2020 Royal Society of Chemistry.

Further investigation into the HDO properties of Ni_2_P/SiO_2_ with guaiacol under varying reaction conditions showed that the reaction pressure significantly influenced the reaction pathways and product distribution [129]. At 1 bar and 300 °C, the direct deoxygenation (DDO) pathway to benzene was favored, achieving 62% selectivity. Conversely, at 8 bar, the prehydrogenation (HYD) pathway led to cyclohexane as the major product, resulting in only 8% benzene selectivity (Figure 11a). This finding aligns with previous studies where cyclohexane was the primary O-free product in the HDO of anisole, emphasizing that low temperatures and higher pressures favor ring saturation [130]. X-ray Absorption Fine Structure (XAFS) measurements and density functional theory (DFT) calculations indicated that both the atomic hydrogen (H) and hydroxyl (OH) groups could adsorb on the unsaturated triple hollow (TFH) Ni sites. These results suggest that the DDO pathway is facilitated by surface OH groups, whereas the HYD pathway is promoted by more reduced surfaces. Consequently, the selectivity for BTX products can be modulated by adjusting the balance of H and OH groups on the catalyst surface.

Additionally, variations in product distribution may also result from different active sites on the Ni_2_P phases and their adsorption behaviors [131,132]. Ni_2_P crystals, characterized by a rhombohedral structure, comprise two types of nickel sites: Ni(1) and Ni(2) [133]. The Ni(1) site, with a sub-tetrahedral structure terminated by Ni_3_P_2_, serves as an active site for H_2_ dissociation and can adsorb phenolic molecules through the oxygen atom of the C-OH bond [132]. After a nucleophilic attack by hydride species, the adsorbed C-OH bonds are cleaved, forming aromatic products (Figure 11b). In contrast, the Ni(2) site may facilitate the flat adsorption of phenolic substrates, promoting hydrogenation of the aromatic rings.

In recent years, Lan and colleagues have further studied the performance of the Ni_2_P/SiO_2_ catalyst in HDO reaction, specifically investigating the removal of cinnamic acid and cresol by the catalyst under 300 °C and 1-bar conditions [134]. The results are encouraging, as it was found that the selectivity of cinnamic acid and cresol for the oxidation of benzene reached 86% and 81%, respectively. However, as the reaction time prolongs, the selectivity of benzene gradually decreases, while the selectivity of phenol significantly increases. This change is closely related to the changes in the surface properties of the catalyst. Specifically, the content of Ni^δ+^ and Ni^0^ on the catalyst surface increases, while the P/Ni ratio decreases. Research suggests that Ni^δ+^, as a Lewis acid site, tends to catalyze demethylation reactions rather than demethoxylation reactions, while Ni^0^ only exhibits low levels of dehydroxylation activity. In addition, the decrease in the P/Ni ratio reduces the number of Brønsted acid sites, thereby inhibiting the dehydroxylation reaction. These changes resulted in a transition from a phosphorus-rich environment on the catalyst surface to a nickel-rich environment, causing the HDO product of cinnamic acid to change from benzene to phenol.

In addition to the Ni_2_P catalyst, the Fe_2_P single-metal catalyst also exhibits high selectivity for cresol in HDO reactions [135]. At 200 °C and atmospheric hydrogen conditions, Fe_2_P can convert cresol to benzene, ultimately achieving a benzene yield of 96% (Figure 11c). It is worth noting that even when the conversion rate is increased to 97.3%, the high selectivity of benzene remains unchanged, indicating that Fe_2_P has unique advantages in Caryl-O bond cleavage. In-depth DFT calculations support this observation, indicating that the oxygen atoms in cresol form a stable conformation with the Fe atoms on the Fe_2_P surface, making the breaking of Caryl-OCH_3_ bonds thermodynamically more favorable (Figure 11d). The calculation results show that compared with other possible cleavage pathways, the adsorption energy for Caryl-OCH_3_ bond cleavage is higher, at −2.9 eV. This further confirms the superiority of Fe_2_P in promoting specific bond cleavage.

The specific mechanism of the reaction can be summarized as follows: the phenol molecule first adsorbs on the Fe_2_P surface, and the benzene ring and oxygen atoms form coordinated bonds with the Fe sites on the surface. Subsequently, the Caryl-O bond is broken, resulting in the formation of C_6_H_5_* and CH_3_O* intermediates. These intermediates further bind with hydrogen atoms dissociated from adjacent Fe sites, ultimately forming benzene. This study not only deepens the understanding of iron-based phosphide catalysts but also broadens the application scope of transition metal phosphides in HDO reactions, providing new ideas for future catalyst designs.

Transition metal phosphide catalysts, such as nickel phosphide (Ni_2_P) and molybdenum phosphide (MoP), have shown remarkable potential in the hydrodeoxygenation (HDO) of phenolic compounds [59,136]. Their superior catalytic activity and selectivity compared to traditional catalysts can be attributed to their unique electronic properties. Moreover, these phosphide catalysts demonstrate higher selectivity towards aromatic compounds, likely due to their ability to stabilize specific intermediates and provide active sites that favor aromatic product formation [137]. However, their poor stability and tendency to deactivate in aqueous environments pose significant challenges for industrial applications.

#### 2.4.3. Transition Metal Carbides, Nitrides

Transition metal carbonitrides exhibit noble metal-like catalytic behavior in the field of directed deoxygenation of phenolic compounds due to their unique electronic structural properties [138,139]. In this type of material, the insertion of carbon/nitrogen atoms into the metal lattice triggers a d-band contraction effect, resulting in an increase in the metal bond length and an increase in the surface electron density, thereby optimizing the C-O bond activation ability [140]. Although the selective regulation law of BTX in this system has not been fully elucidated, the groundbreaking work of Lee’s team has confirmed that the Mo_2_C catalyst can achieve the efficient conversion of benzyl ether under normal temperature and pressure conditions, with BTX selectivity exceeding 90% [141]. Its superiority lies in the selective passivation of hydrogenation sites by oxygen-containing intermediates generated in situ, which has been further validated in the guaiacol conversion system (benzene selectivity >90%) [142].

Reaction pathway analysis reveals that the lower dissociation energy of the Caryl-OCH_3_ bond compared to Caryl-OH promotes the preferential removal of methoxy groups [142]. Synchronous characterization confirmed that phenolic compounds form strong electronic coupling with the Mo_2_C surface through Caryl-O bonds, and this specific adsorption configuration promotes weaker CarylO-CH_3_ bonds to preferentially break over C-OH bonds. In addition, methanol/water titration experiments showed that the dynamic modification of surface-oxygen-containing species can inhibit aromatic ring hydrogenation activity, increasing aromatic selectivity [122].

In recent years, in-depth research has been conducted on the surface chemical properties of Mo_2_C catalysts in HDO reactions, especially when using the operating state NAP-XPS technology [143]. It was found that the Mo^3+^ phase on the surface of Mo_2_C is dominant, and there is also a small amount of high-valence molybdenum ions (Mo^5+^ and Mo^6+^) on the surface. It is worth noting that even in the presence of oxygenated compound feeds, the oxidation state and oxygen coverage of molybdenum remain constant throughout the entire reaction process. Meanwhile, the dominant position of Mo^2+^ and the slight changes in its high-valence-state molybdenum ions (Mo^5+^/Mo^6+^) indicate that the carbonyl phase is the main active site of Mo_2_C in the HDO reaction.

In this regard, Wang et al. further investigated the performance of a Mo_2_C-MoC_x_O_y_ composite catalyst on activated carbon for the HDO reaction of meta-methylphenol [144]. At a 623 K and 4.3 MPa H_2_ pressure, the catalyst can selectively generate toluene with 75% selectivity. Through detailed study of kinetics, it was found that catalysts with different Mo_2_C-MoC_x_O_y_ ratios exhibit consistent apparent activation energies on the direct oxidation and hydrogenation reaction pathways. This indicates that the active sites of the catalyst are determined by their own state and may be related to the adsorption or exchange of oxygen on the catalyst surface. From this, it can be inferred that both the Mo_2_C and MoC_x_O_y_ phases may promote the oxidation reaction.

In addition, the ordered mesoporous Mo_2_C and W_2_C catalysts were prepared by the hard-template method, and their performance under reaction conditions also varied depending on the type of metal [145]. At atmospheric pressure and 443 K, the selectivity of the W_2_C catalyst for converting cresol to benzene reached 96%, while the selectivity of the Mo_2_C catalyst for benzene was slightly lower at 423 K, at 80%. This difference is mainly attributed to the higher oxide affinity of tungsten compared to that of molybdenum. Dynamics studies have shown that the production rate of benzene catalyzed by W_2_C and Mo_2_C is almost independent of the pressure of cresol, but the dependence on H_2_ pressure is close to half order, indicating that the generation of benzene requires two different active sites. Although the apparent activation energies of the two catalysts are similar, the formation frequency of benzene catalyzed by W_2_C is only 20% of that of Mo_2_C, indicating that the essential differences in the epitopes of tungsten carbide and molybdenum are not determined by the identity of the epitopes. This may be because the excessively strong W-O bond reduces the oxidation efficiency, thereby affecting the rate of benzene formation.

**Figure 12:**
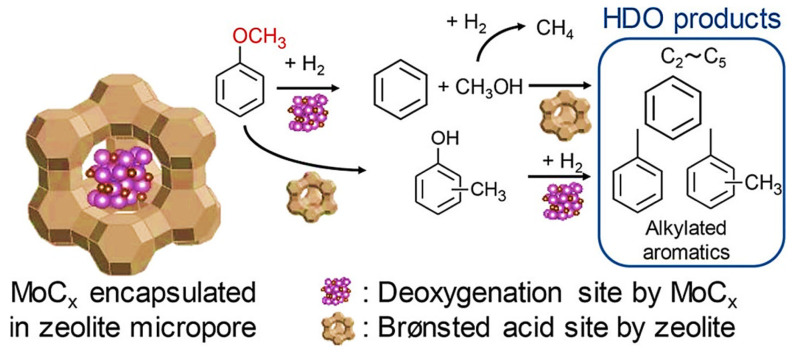
The HDO of anisole to BTX by a MoC_x_-encapsulated FAU zeolite catalyst [146]. Copyright 2017 American Chemical Society.

The dispersion of MoC_x_ nanoclusters into the micropores of faujasite zeolite (FAU) also significantly improved the stability of the catalyst [146]. The catalyst prepared by this strategy can promote an alkylation reaction with high aromaticity selectivity, thereby retaining carbon in the desired final product (Figure 12). This high selectivity is mainly attributed to the Brønsted acid sites in the MoC_x_/FAU catalyst transferring the methoxy group of one molecule of cresol to another molecule. Compared with the physical mixture of Mo_2_C and FAU, the bifunctional reaction stability of MoC_x_/FAU is significantly improved within 20 h. This is because in the Mo_2_C + FAU mixture, the physical distance between the Brønsted acid site and the Mo_2_C site is relatively long, resulting in the rapid desorption of intermediates before continuous reaction steps, thereby forming high BTX selectivity [146]. This discovery not only demonstrates the important advantages of introducing transition metal carbonyl nanoclusters into zeolite structures but also opens up new pathways for other chemical reactions that require bifunctional metals and acid sites. In addition to gas-phase reactions, MoC_x_ is also used in liquid-phase reactions. The Smirnov team has developed MoC_x_-SiO_2_ and bimetallic NiMoC_x_-SiO_2_ catalysts for the HDO reaction of cresol [147]. At 320 °C and 60-bar pressure, the MoC_x_-SiO_2_ catalyst can selectively convert cresol to benzene with approximately 70% selectivity. Dynamics studies have shown that the reaction pathway involves direct cleavage of C-O bonds to form benzene, rather than through phenolic intermediates. Meanwhile, the selectivity of benzene depends on the variation in the Ni content. The use of Ni as a promoter increases the activity of ring saturation and reduces the selectivity of bimetallic catalysts for benzene.

Recent advancements reveal that constructing bimetallic carbide systems (e.g., Mo-W carbides) significantly enhances the catalytic performance in lignin valorization. Tran’s team conducted a pioneering comparison between MoWC composites and their monometallic counterparts (Mo_2_C, WC) for guaiacol hydrodeoxygenation (HDO) [148]. The experimental data demonstrated the superior activity of MoWC, achieving high benzene selectivity at 92% conversion, starkly contrasting with WC’s mere 23% conversion. Notably, product profiles diverged substantially: Mo_2_C predominantly yielded cyclohexene (59.1%), whereas WC favored phenol formation (69%). This performance disparity originates from the dual-active-site configuration in MoWC systems’ oxygen-affinic centers and hydrogen-activation sites [149]. The incorporation of tungsten enhances surface oxophilicity, preferentially adsorbing oxygen-containing moieties (hydroxyl/methoxy groups) over aromatic π-electrons, thereby suppressing cycloaddition side reactions. XPS characterization confirmed electron migration from W to Mo atoms, inducing distinct electronic configurations compared to monometallic carbides. The synergistic interplay in MoWC enables effective H_2_ activation while maintaining strong reactant adsorption through oxygen functionalities, collectively promoting selective oxygen removal during HDO processes [148].

Some research has investigated nitride catalysts, such as MoN_x_ and CoMoN_x_, using guaiacol as a typical model compound to partially deoxygenate it to other phenols [150,151]. However, this makes it a challenge to directly compare BTX selectivity. Likewise, the group of Ga-synthesized Mo_2_N and CoMoN_x_ catalysts in the HDO of guaiacol [152]. These nitriles directly cleave the Caromatic–OCH_3_ bond in guaiacol to form phenol and then saturate the ring in the HDO process to form cyclohexene and cyclohexane at 300 °C and 50-bar H_2_ pressure. Notably, the product distribution can be adjusted by applying the oxophilic support. As an example, when Mo_2_N is loaded on TiO_2_, the catalyst exhibits more than 90% BTX selectivity in phenol HDO at 623 K and 2.5 MPa [153]. The high activity might be attributed to TiO_2_ as an oxygenophilic support that promotes the selective cleavage of Caryl-O bond similarly to metal catalysts. Therefore, further in-depth studies on it are needed in the future.

In general, carbide and nitride catalysts, including molybdenum carbide (Mo_2_C), exhibit promising catalytic performance in the hydrodeoxygenation (HDO) of phenolic compounds. However, these catalysts face limitations such as susceptibility to oxidation, which compromises their stability. Additionally, the reaction mechanisms underlying their catalytic activity remain poorly understood. To address these challenges, further investigations are required to enhance the stability of carbide catalysts and elucidate their reaction mechanisms.

## 3. Conclusions and Perspectives

With the increasing demand for fuels and the heavy reliance on non-renewable petroleum, the sustainable production of chemicals and fuels from renewable resources has emerged as a critical scientific and engineering objective for modern society. Substantial global research efforts are focused on identifying and developing renewable energy sources to replace traditional fossil feedstocks in our current energy framework. Among various biomass constituents, lignin stands out as a particularly promising candidate due to its unique aromatic architecture and high energy density [39,154,155]. The catalytic hydrodeoxygenation (HDO) of lignin-derived phenolics has emerged as a transformative approach for generating value-added aromatic commodities, offering a sustainable pathway to mitigate fossil resource depletion [156,157,158,159]. This review systematically examines recent breakthroughs in the catalytic transformation of representative lignin-derived phenolic compounds into functionalized aromatics, including benzene and its derivatives. And the conversion rates and selectivity of different catalysts are shown in Table 1. Nevertheless, several fundamental challenges persist, encompassing both technological limitations and mechanistic uncertainties, which must be resolved to advance this field toward practical implementation.

(i) Catalyst deactivation and stability remain critical challenges in HDO transformations, significantly impacting industrial applicability. Common deactivation mechanisms include coking (accumulation of polyaromatic species on active sites), metal sintering (particle agglomeration under high temperature/hydrogen pressure), and oxidation (loss of active metal states in oxygen-containing environments). Stability issues are further exacerbated in aqueous-phase HDO due to metal–support interactions and hydrolysis. Future research must prioritize (1) developing anti-coking catalyst architectures (e.g., mesoporous supports, core–shell structures) to mitigate pore blockage, (2) engineering robust metal–support interfaces (e.g., strong metal–support interactions, oxide-stabilized metal nanoparticles) to suppress sintering, (3) exploring redox-active additives to maintain metal reducibility and oxygen vacancy stability, and (4) implementing in situ regeneration protocols (e.g., periodic oxidation–reduction cycles) to restore catalyst activity. Addressing these challenges will be pivotal for translating lab-scale HDO efficiency into industrially viable, long-duration catalytic processes.

(ii) Green solvent engineering in phenolic hydrogenation–deoxygenation processes. In modern sustainable chemistry, the development of eco-friendly reaction media represents a critical challenge for industrial applications. Water has been widely validated as a green reaction medium for the selective HDO of phenolic compounds, offering advantages such as high activity and product specificity. However, its inherent volatility at reaction temperatures (typically >150 °C) leads to significant solvent loss and corrosion risks in reactor systems. To address these limitations, recent research has explored the use of low-volatility solvents with tunable polarity. Ionic liquids (ILs), with their customizable anion–cation interactions, have shown potential for improving product selectivity through solvation effects. Concurrently, deep eutectic solvents (DESs) composed of hydrogen bond donors and acceptors have emerged as promising alternatives, exhibiting a comparable catalytic performance to that of ILs while offering lower toxicity and higher biodegradability. Despite these advances, current solvent optimization studies are primarily empirical, lacking systematic mechanistic analysis linking solvent properties to reaction outcomes. Therefore, computational modeling combined with in situ characterization techniques should be prioritized to establish structure–activity relationships, enabling rational designs of next-generation solvent systems.

(iii) The current state of the art in phenolic HDO catalysis relies predominantly on miniaturized steel reactors or batch-type systems for catalyst development and kinetic studies, leveraging their ease of operation and process simplicity. While these platforms enable high-throughput catalyst evaluation and kinetic parameter determination, large-scale industrial implementation necessitates fixed-bed reactors due to their superior continuous processing capabilities and potential for higher space–time yields. To bridge the gap between lab-scale success and commercial deployment, future research should focus on process intensification strategies for continuous-flow systems, integrating catalyst design with reactor engineering. This approach would allow precise control over residence time distribution, reactant concentration gradients, and thermal management, thereby improving product selectivity. However, scaling up HDO processes faces significant challenges, including heterogeneous reaction dynamics at biomass–catalyst interfaces, multiphase mass-transfer limitations, and reactor fouling mitigation. Computational fluid dynamics (CFD) modeling combined with in situ characterization techniques will be essential for optimizing reactor hydrodynamics and heat-transfer coefficients. Additionally, techno-economic analysis should be conducted to evaluate the feasibility of integrating HDO units with existing biorefinery infrastructure, ensuring compatibility with downstream processing operations. The current literature lacks comprehensive studies on reactor performance stability over extended operation periods, which is critical for industrial viability.

(iv) Considering the biomass-derived nature of phenolic compounds, future research should shift focus toward valorizing lignin as a sustainable feedstock for producing platform chemicals and renewable fuels. Central to this effort is the design of one-pot catalytic systems that couple lignin depolymerization with in situ hydrogenation–deoxygenation (HDO), enabling the direct conversion of native lignin into high-value products. Such integrated processes offer dual advantages: minimizing post-depolymerization stabilization steps while reducing energy-intensive separation operations through enhanced product selectivity. However, realizing this potential requires resolving fundamental gaps in understanding lignin’s structural transformation during HDO, particularly regarding its macromolecular architecture, solubility behavior, and thermal stability. Recent advancements in advanced characterization tools, including heteronuclear single-quantum coherence (HSQC) spectroscopy, three-dimensional nuclear magnetic resonance (3D NMR), and long-range correlation (HMBC) techniques, have enabled unprecedented insights into lignin’s chemical composition. Complementary in situ techniques, such as synchrotron-based X-ray absorption spectroscopy (XAS) and time-resolved infrared (TRIR) spectroscopy, are now being employed to monitor dynamic structural changes during HDO. The thermodynamic modeling of lignin-derived phenolic intermediates and their HDO pathways is also gaining traction, with computational methods like density functional theory (DFT) providing atomistic-level mechanistic insights. Future breakthroughs in this field will likely arise from integrating experimental characterization with multi-scale modeling approaches to design tailored catalysts capable of selectively targeting lignin’s recalcitrant linkages while maintaining high stability under industrially relevant conditions.

In conclusion, this review aims to offer a thorough understanding of the current state of knowledge regarding the production and innovative catalytic upgrading of functional chemicals. These insights are applicable to the catalytic hydrodeoxygenation (HDO) upgrading pathways of various biomass-derived platform model compounds, whether originating from lignin or carbohydrates. Furthermore, the integrated approach combining lignin depolymerization with downstream processes such as HDO and etherification (ECH) presents a promising pathway for the sustainable production of high-value functional chemicals in the future. This research field is bound to greatly expand and provide a green future for our society with sustainable chemical products.

## Figures and Tables

**Table 1:** Performances of different catalysts with BTX selectivity in the HDO of lignin-derived phenolic compounds.

Catalyst	Reaction Condition				Substrate	Conv. (%)	Products	Sel. (%)	Ref.
	T (°C)	P (bar)	Solvent	Reactor					
Pd/Fe_2_O_3_	300	~1.01	--	fixed-bed	m-cresol	~55	BTX	94	[43]
Pd/Nb_2_O_5_	300	1	--	fixed-bed	phenol	6.6	benzene	80.2	[44]
Pd/TiO_2_	300	1	--	continuous-flow	phenol	7.0	benzene	66.8	[46]
Pd/ZrO_2_	300	1	--	fixed-bed	phenol	77	benzene	66	[160]
Pd/Nb_2_O_5_	300	1	--	fixed-bed	m-cresol	13.1	toluene	95.6	[47]
Pd/TiO_2_	300	1	--	fixed-bed	m-cresol	7.3	toluene	80.0	[47]
Pd/ZrO_2_	300	1	--	fixed-bed	m-cresol	14.7	toluene	87.9	[47]
Pd/t-ZrO_2_	300	1.01	--	fixed-bed	phenol	86	benzene	44.2	[161]
Pd/SiO_2_-Al_2_O_3_	450	1	--	continuous-flow	guaiacol	83	BTX	73	[162]
Ru/Nb_2_O_5_	250	5	water	fixed-bed	p-cresol	100	toluene	80	[48]
Ru/Nb_2_O_5_	250	5	water	fixed-bed	cresol	100	BTX	80	[48]
Ru/Nb_2_O_5_-MC	250	2	decalin/water mixed	batch	phenol	100	benzene	80	[51]
Ru/Nb_2_O_5_-SiO_2_	230	--	2-PrOH	batch	p-cresol	98.5	toluene	84.0	[52]
Ru/TiO_2_	300	37.9	water as additive	batch	phenol	30	benzene	95	[53]
Ru/TiO_2_	300	10	decane	batch	anisole	81	benzene	79	[86]
Ru/TiO_2_	200	5	decane	batch	anisole	41	benzene	50.2	[163]
Ru/TiO_2_ +N_2_	220	1	decalin	batch	p-cresol	97.4	toluene	98.4	[54]
Ru/HZSM-5	240	2	water	batch	guaiacol	100	benzene	95	[55]
Ru/HZSM-5	240	2	water	batch	anisole	100	benzene	94.4	[55]
Ru/HZSM-5	240	2	water	batch	phenol	100	benzene	100	[55]
Ru/HZSM-5	240	2	water	batch	4-methylguaiacol	100	toluene	93	[55]
Ru/HZSM-5	240	2	water	batch	syringol	100	toluene	95	[55]
Ru/HZSM-5	240	2	water	batch	syringol	100	benzene	94	[164]
Ru/LaCO_3_OH	240	2	water	batch	guaiacol	95.6	benzene	75.8	[165]
Ru/C	400	40	--	fixed-bed	guaiacol	100	benzene	69.5	[166]
Ru/MoO_x_-ZrO_2_	250	10	deionized water	steel autoclave	anisole	77.6	benzene	84.7	[56]
Pt/Hbeta	400	1	--	continuous-flow	cresol	100	BTX	>90	[57]
Pt/Hbeta	400	1	--	continuous-flow	anisole	100	BTX	90	[58]
Pt/Hbeta	350	1	--	continuous-flow	guaiacol	90	BTX	85	[60]
Pt/SiO_2_	400	1	--	continuous-flow	cresol	91	toluene	>75	[57]
Pt/SiO_2_	400	1	--	continuous-flow	anisole	100	BTX	69	[58]
Pt/TiO_2_	300	1	--	continuous-flow	cresol	17	toluene	88	[167]
Pt/TiO_2_	350	5	--	packed-bed	m-cresol	35	toluene	78	[61]
Pt/ZrO_2_	300	1	--	continuous-flow	cresol	12	toluene	67	[167]
Pt/UiO-66-def	270	10	decane	fixed-bed	anisole	70–86	benzene	52	[168]
Pt/C	350	5	--	packed-bed	m-cresol	35	toluene	46	[61]
MoO_3_	325	1	--	continuous-flow	cresol	48.9	BTX	99.4	[66]
MoO_3_	340	35	octane	batch	phenol	98.1	benzene	99.5	[62]
MoO_3_	340	35	octane	batch	anisole	98.2	BT	78.3	[62]
MoO_3_	340	35	octane	batch	guaiacol	98.0	BT	82.7	[62]
MoO_3_	340	35	octane	batch	cresol	78.9	BT	98.5	[62]
MoO_3_-SiO_2_	340	5	--	fixed-bed	phenol	89.6	benzene	84.3	[169]
MoO_3_-SiO_2_	340	5	--	fixed-bed	p-cresol	90.7	toluene	81.2	[169]
MoO_3_-SiO_2_	340	5	--	fixed-bed	anisole	88.5	BT	76.0	[169]
MoO_3_/ZrO_2_	320	1	--	continuous-flow	cresol	78	toluene	77	[67]
MoO_3_/TiO_2_	320	1	--	continuous-flow	cresol	47	toluene	46	[67]
MoO_3_/CeO_2_	320	1	--	continuous-flow	cresol	8	toluene	7	[67]
MoO_3_/Al_2_O_3_	320	1	--	continuous-flow	cresol	13	toluene	10	[67]
MoO_3_/SiO_2_	320	1	--	continuous-flow	cresol	10	toluene	9	[67]
MoO_3_/TiO_2_	350	25	--	continuous-flow	phenol	28	benzene	89	[153]
MoO_x_/SiO_2_	340	40	--	continuous-flow	cresol	23.8	benzene	81.9	[68]
MoO_x_/SBA-15	340	40	--	continuous-flow	cresol	23.9	benzene	82.8	[68]
MoO_x_/Al_2_O_3_	340	40	--	continuous-flow	cresol	22.1	benzene	85.5	[68]
Ni/CeO_2_	290	3	--	continuous-flow	anisole	88	benzene	55	[74]
Ni/TiO_2_	290	3	--	continuous-flow	anisole	51	benzene	74	[74]
Ni/C	290	3	--	continuous-flow	anisole	95	benzene	51	[74]
Ni/SBA-15	290	3	--	continuous-flow	anisole	99	benzene	8	[74]
Ni/Al_2_O_3_	290	3	--	continuous-flow	anisole	99	benzene	27	[74]
Ni/SiO_2_	300	1	methanol	continuous-flow	phenol	90	benzene	99	[170]
Ni/Al_2_O_3_	260	1	--	continuous-flow	cresol	30	toluene	67.8	[171]
Ni/Ce_0.3_Nb_0.7_O_2_	300	1	--	continuous-flow	phenol	9.53	benzene	86.92	[77]
Ni/Nb_2_O_5_	300	1	--	continuous-flow	phenol	10.10	benzene	89.96	[77]
Ni@silicalite-1	250	2.5	n-decane	fixed-bed	m-cresol	78.4	BT	73.1	[63]
Fe_2_O_3_	300	1	--	continuous-flow	cresol	21	BTX	90	[43]
Fe	300	1	--	continuous-flow	m-cresol	1–10	toluene	>90	[172]
Fe/NC-3.7	350	5	--	fixed-bed	m-cresol	20.1	toluene	78.2	[173]
Fe/SiO_2_	375	1	--	continuous-flow	anisole	8	benzene	85	[174]
Fe/SiO_2_	300	1	--	continuous-flow	m-cresol	8.8	toluene	60.2	[175]
Fe/SiO_2_	400	1	--	continuous-flow	guaiacol	100	BT	>90	[176]
Cu/MnAlO_x_	300	20	n-decane	stainless autoclave	anisole	54	benzene	65	[78]
PdFe/C	450	1	--	continuous-flow	guaiacol	100	benzene	83.2	[84]
RuFe/meso-TiO_2_	250	1	decane	batch	anisole	98	benzene	>80	[86]
FeReO_x_/ZrO_2_	350	1	--	batch	anisole	100	BTX	48.3	[26]
FeReO_x_/ZrO_2_	350	1	--	batch	m-cresol	100	BTX	61.7	[26]
FeReO_x_/ZrO_2_	350	1	--	batch	guaiacol	100	BTX	21.6	[26]
FeReO_x_/MCM-41	350	1	--	batch	anisole	100	BTX	25.8	[26]
FeReO_x_/MCM-41	350	1	--	batch	m-cresol	100	BTX	29.6	[26]
FeReO_x_/MCM-41	350	1	--	batch	guaiacol	100	BTX	8.0	[26]
FeReO_x_/HBeta	350	1	--	batch	m-cresol	100	BTX	42.4	[26]
1Pt@-10Fe@SiO_2_	450	5	--	fixed-bed	guaiacol	96.3	BTX	48.8	[88]
NiFe/SiO_2_	300	1	--	continuous-flow	cresol	13.7	toluene	52.6	[65]
NiMo/SiO_2_	410	1	--	continuous-flow	phenol	99.3	benzene	99.27	[91]
NiMo/SiO_2_	410	1	--	continuous-flow	anisole	99.35	BTX	98.55	[91]
NiMo/SiO_2_	410	1	--	continuous-flow	guaiacol	99.79	BTX	97.53	[91]
NiMo/SiO_2_	350	1	--	continuous-flow	m-cresol	100	toluene	>80	[92]
Ni_40_In/SiO_2_	300	1	--	fixed-bed	anisole	97	BTX	60.4	[93]
NiGa/SiO_2_	300	1	--	fixed-bed	anisole	31	benzene	92.6	[96]
PB-Hβ-8/Ni-V	350	1	--	fixed-bed	guaiacol	100	BT	69.17	[97]
NiRe/SiO_2_	300	1.01	--	fixed-bed	m-cresol	47.6	toluene	50	[95]
NiReO_x_/ZrO_2_	350	1	--	continuous-flow	m-cresol	63.9	BTX	90	[99]
Ni-ReO_x_/CeO_2_	350	1	--	continuous-flow	m-cresol	45.7	BTX	83	[99]
Ni-ReO_x_/ZrCeO_2_	350	1	--	continuous-flow	m-cresol	42.5	BTX	80	[99]
PdReO_x_/ZrO_2_	350	1	--	continuous-flow	m-cresol	80	BTX	77.2	[99]
Re-MoO_x_/TiO_2_	300	30	dodecane	batch	anisole	10	BTX	~50	[101]
Re-VO_x_/TiO_2_	300	30	dodecane	batch	anisole	10	BTX	~56	[101]
Re-MoO_x_/TiO_2_	300	50	dodecane	batch	anisole	35	benzene	83	[100]
PtWO_x_/C	300	36	dodecane	fixed-bed	m-cresol	61	toluene	98	[103]
RuW/SiO_2_	175	5	water	batch	anisole	100	benzene	99.2	[104]
RuWO_x_/SiAl	220	10	water	batch	phenol	100	benzene	77	[102]
RuWO_x_/ZrO_2_	220	10	water	batch	phenol	100	benzene	65	[102]
Pt-Mo/CNT	300	1	--	continuous-flow	dihydroeugenol	100	BTX	93.2	[176]
PtSn/CNF/Inconel	400	1	--	continuous-flow	anisole	74	benzene	80	[177]
CoMoS	300	40	decalin	batch	guaiacol	100	benzene	86	[112]
CoMoS	300	40	decalin	batch	phenol	100	benzene	100	[112]
CoMoS	300	40	decalin	batch	cresol	100	benzene	100	[112]
CoMoS	300	40	--	fixed-bed	guaiacol	50	benzene	42	[178]
CoMoS-0.18	250	30	decalin	sealed autoclave	p-cresol	92.4	toluene	95.5	[113]
Co-MoS_2_-2	250	30	decalin	autoclave	p-cresol	92.7	toluene	94.1	[114]
CoMoS/Al_2_O_3_	340	40	dodecane	batch	m-cresol	19.6	toluene	28	[179]
CoMoS/Al_2_O_3_	300	15	xylene	continuous-flow	phenol	71.9	benzene	86.4	[180]
CoMoS/Al_2_O_3_	300	15	xylene	continuous-flow	anisole	96.8	BT	32.5	[180]
CoS_2_/MoS_2_	250	40	dodecane	batch	p-cresol	98	toluene	99	[181]
Mo_0.06_-Co_9_S_8_/Al_2_O_3_	265	30	methylcyclohexane	batch	phenol	95.7	benzene	89.6	[115]
Mo_0.06_-Co_9_S_8_/Al_2_O_3_	265	30	methylcyclohexane	batch	p-cresol	98.2	toluene	89.2	[115]
Mo_0.06_-Co_9_S_8_/Al_2_O_3_	265	30	methylcyclohexane	batch	guaiacol	99.5	BT	71.0	[115]
Mo_0.06_-Co_9_S_8_/Al_2_O_3_	265	30	methylcyclohexane	batch	anisole	99.7	benzene	80.1	[115]
Mo_0.06_-Co_9_S_8_/Al_2_O_3_	265	30	methylcyclohexane	batch	catachol	99.8	benzene	72.4	[115]
Co-MoS_2-x_	200	40	dodecane	batch	4-methylphenol	97.4	toluene	99.6	[116]
MoS_2_	300	40	dodecane	batch	p-cresol	67.0	toluene	74.1	[116]
NiS_2_	300	40	dodecane	batch	p -cresol	32.4	toluene	55.6	[116]
Ni-S+MoS_2_	300	40	dodecane	batch	p -cresol	59.2	toluene	84.6	[119]
Mo-W-S	300	30	decalin	batch	4-methylphenol	50	toluene	89	[182]
Ni-Mo-W-S	300	30	decalin	batch	4-methylphenol	100	toluene	99.4	[182]
MoP/SiO_2_	300	1.01	--	continuous-flow	guaiacol	54	benzene	53	[178]
MoP/TiO_2_	350	25	decane	continuous-flow	phenol	25	benzene	82	[153]
MoP-CA	500	44	decalin	batch	4-methylphenol	58	toluene	60	[183]
Co_2_P/SiO_2_	300	1.01	--	continuous-flow	guaiacol	70	benzene	52	[178]
Fe_2_P	200	1.01	--	batch	anisole	100	benzene	96.7	[135]
FeMoP	400	21	decalin	batch	phenol	>99	benzene	~90	[123]
FeMoP	400	21	decalin	batch	anisole	90	benzene	92	[123]
Ni_2_P/SiO_2_	300	1.01	--	continuous-flow	guaiacol	80	benzene	60	[178]
Ni_2_P/SiO_2_	300	1.4	--	fixed-bed	guaiacol	>99	benzene	72	[184]
Ni_2_P/SiO_2_	300	10	--	fixed-bed	guaiacol	64	BTX	60	[126]
Ni_2_P/SiO_2_	300	1	tridecane	continuous-flow	guaiacol	85	benzene	62	[129]
Ni_2_P/SiO_2_	400	15	--	continuous-flow	anisole	100	benzene	60.6	[130]
Ni_2_P/SiO_2_	300	1	--	continuous-flow	guaiacol	55	benzene	86	[131]
Ni_2_P/SiO_2_	300	1	--	continuous-flow	anisole	71	benzene	81	[134]
Mo_2_C	350	27.6	decane	batch	guaiacol	78	benzene	22.1	[148]
Mo_2_C	250	1.013	--	fixed-bed	anisole	49	benzene	87	[146]
β-Mo_2_C	350	4.4	--	continuous-flow	guaiacol	99.8	benzene	87.8	[185]
Mo_2_C	60	10	--	continuous-flow	m-cresol	21	toluene	95	[186]
Mo_2_C	150	1.31	--	fixed-bed	anisole	40	benzene	~80	[145]
Mo_2_C	130	1	--	continuous-flow	anisole	100	benzene	>90	[141]
Mo_2_C	280	1.1	--	continuous-flow	phenolic mixture	94	BT	93	[142]
Mo_2_C/TiO_2_	350	25	decane	continuous-flow	phenol	65	benzene	91	[153]
Mo_2_C/CNF	350	55	--	batch	guaiacol	45	BT	2	[187]
Mo_2_C+FAU	250	1.013	--	fixed-bed	anisole	61	BT	40	[146]
MoC_x_/FAU	250	1.013	--	fixed-bed	anisole	97	BT	10	[146]
MoC-SiO_2_	32	60	hexadecane	batch	anisole	65	benzene	~70	[147]
Mo_2_C-MoC_x_O_y_	350	43	decalin	batch	p-cresol	25	toluene	>75	[144]
W_2_C	171	1.31	--	fixed-bed	anisole	100	benzene	96	[145]
W_2_C/CNF	350	55	--	batch	guaiacol	66	BT	1	[187]
NiMoC-SiO_2_	32	60	hexadecane	batch	anisole	95	benzene	20	[147]
MoWC	350	27.6	decane	batch	guaiacol	93	BT	76.4	[148]
Mo_2_N/TiO_2_	350	25	decane	continuous-flow	phenol	9.1	benzene	90	[153]
